# Synchronous Smiles and Hearts: Dyadic Meditations Enhance Closeness and Prosocial Behavior in Virtual and In-Person Settings

**DOI:** 10.1007/s12671-025-02588-7

**Published:** 2025-05-19

**Authors:** Vera.U. Ludwig, Lana Prieur, Scott M. Rennie, Andrew Beswerchij, Devora Weintraub, Blaire Berry, Jenny Wey, Katelyn Candido, Michael L. Platt

**Affiliations:** 1 Department of Neuroscience, Perelman School of Medicine, University of Pennsylvania, Philadelphia, USA; 2 Wharton Neuroscience Initiative, the Wharton School of Business, University of Pennsylvania, Philadelphia, USA; 3 Positive Psychology Center, University of Pennsylvania, Philadelphia, USA; 4 Champalimaud Centre for the Unknown, Champalimaud Foundation, Lisbon, Portugal; 5 Department of Marketing, McCombs School of Business, University of Texas, Austin, USA; 6 Department of Psychological Science, Pomona College, Claremont, USA; 7 Department of Psychology, School of Arts and Sciences, University of Pennsylvania, Philadelphia, USA

**Keywords:** Relationships, Connection, Synchrony, Loneliness, Remote

## Abstract

**Objectives:**

Social connection is crucial for well-being and health. Dyadic meditations—contemplative practices carried out by two people together—have the potential to foster connection. In the dyadic “Just-Like-Me” (JLM) meditation, two participants gaze at each other while contemplating sentences emphasizing their shared humanity. We assessed the psychological impacts of this exercise, as well as the underlying mechanisms, by comparing it to two active control conditions: mutual gazing without contemplation and solitary meditation.

**Method:**

Study 1 was a virtual study with 55 individuals who formed 100 experimental dyads, whereas Study 2 was an in-person study with 98 participants in 238 dyad pairings. Participants engaged in a 2-min JLM, gazing, or solitary meditation exercise (the latter only in Study 2). We recorded self-reported feelings, decisions on a hypothetical dictator game, facial expressions (Study 1), and heart rates (Study 2).

**Results:**

Both JLM and gazing increased closeness with medium-to-large effect sizes both virtually and in person (~ 1 *SD* increase for JLM). JLM increased closeness more than gazing in person (medium-sized effect). Both exercises had small-to-medium effects on positive partner perceptions. In-person, dictator game allocations were higher following JLM than following solitary meditation. Both JLM and gazing induced synchronous smiling, with JLM producing stronger effects (Study 1). JLM induced synchronous heart rates (Study 2). Smiling synchrony predicted positive relational outcomes with small-to-medium effect sizes.

**Conclusions:**

Dyadic meditations, such as JLM and gazing, are effective in promoting closeness and prosocial behavior. Non-verbal and emotional synchrony between meditation partners is a potential mechanism facilitating these benefits. Dyadic meditation practices may contribute to addressing widespread loneliness and enhancing social dimensions of well-being.

**Preregistration:**

This study is not preregistered.

**Supplementary Information:**

The online version contains supplementary material available at 10.1007/s12671-025-02588-7.

Human connection is essential for well-being, with good relationships tied to healthier and happier lives (Diener & Seligman, 2002; Yang et al., 2016). Social isolation raises mortality risk more than smoking or obesity (Holt‑Lunstad et al., 2010). Social bonds help people cope with stress, fulfill basic needs, and provide access to resources and information (Baumeister & Leary, 2017; Podolny & Baron, 1997). In the workplace, they increase motivation (Ryan & Deci, 2017), employee retention (De Clercq et al., 2020), and productivity (Friedman et al., 2018). Currently, however, widespread loneliness contributes to high rates of depression, anxiety, and suicide (Na et al., 2023; Office of the Surgeon General, 2023; Sterling & Platt, 2022). The COVID-19 pandemic exacerbated these issues, highlighting the urgent need for new ways to foster connection (Kubzansky et al., 2023).

Dyadic meditations—where two participants meditate in pairs rather than alone—may be one promising approach to address this need (Godara et al., 2024). In contrast to solitary meditation (Kabat‑Zinn, 2003; Teasdale et al., 2000), dyadic meditations involve various forms of interaction, such as verbal exchanges or non-verbal activities like synchronized breathing (Järvelä et al., 2021; Kok & Singer, 2017). Even solitary meditations such as mindfulness can foster prosocial behavior (Berry et al., 2020; Donald et al., 2019), and variants of solitary meditation exist that specifically focus on cultivating prosocial states (Fredrickson et al., 2017; Hofmann et al., 2011). However, dyadic meditations may hold additional promise for improving relationships because they engage processes directly relevant to social interactions, such as listening (Petzold et al., 2023).

Various dyadic meditation methods are used in practice. For instance, *Enlightenment Intensive* retreats incorporate dyadic practices to foster personal and spiritual growth (Berner & Wexler, 2013; Chapman, 1988). The *Global Dyad Meditation* platform utilizes teleconferencing applications to connect individuals worldwide for dyadic meditations (Anliker, 2020). The *Human Connection Project* organizes in-person events where participants silently look into each other’s eyes (The Human Connection Movement, 2021). Moreover, *Insight Dialogue* uses dyadic inquiry within mindfulness courses (Kramer & O’Fallon, 1997).

Singer and colleagues explored dyadic meditation—often abbreviated as *dyads*—in two large-scale studies: the *ReSource* project (Singer, 2016) and the *CovSocial* project (Godara et al., 2021). ReSource included two types of dyads alongside other interventions. In the Affect Dyad, participants shared challenging experiences or experiences they were grateful for while their partner listened attentively without responding. The Perspective Dyad involved participants describing situations from the perspective of an “inner part” (e.g., an inner critic) while the partner listened. Both dyadic exercises increased feelings of closeness and fostered self-disclosure over time (Kok & Singer, 2017). These dyadic exercises also led to more positive affect, especially for Affect Dyads. CovSocial extended this work, showing that regular, virtual dyads—accompanied by virtual coaching sessions—reduced loneliness more than solitary mindfulness meditation accompanied by coaching (Matthaeus et al., 2024). Virtual dyads were also more effective in increasing positive and other-regarding thoughts (Petzold et al., 2023). Schültke et al. (2024) tested an in-person compassion meditation with romantic couples, comparing solitary meditation with partners separated by a partition to a dyadic format with periodic mutual gaze. Both formats increased self-reported closeness and positive affect.

Beyond understanding the outcomes of dyadic exercises, it is imperative to investigate their underlying mechanisms, which can help validate the interventions and inform their improvement. Addressing the affective-cognitive level, Godara et al. (2024) showed that their online program involving Affect Dyads in CovSocial reduced negative interpretation biases (the tendency to interpret ambiguous stimuli as negative), which, in turn, was associated with reduced post-intervention psychopathology. Moreover, increased acceptance of difficult situations after the program predicted increases in self-compassion (Silveira et al., 2023). On the bio-behavioral level, increased activation and structural plasticity in brain regions associated with socio-emotional processing may play a role in mediating the beneficial effects of dyads and related practices (Böckler et al., 2018; Valk et al., 2017). Engagement of socio-emotional brain circuitry in two or more people simultaneously can lead to increased interpersonal synchrony (Kinreich et al., 2017). Synchrony is defined as the temporal alignment of physiological or behavioral processes between individuals. It has, for example, been observed in brain activity, pupillary responses, facial expressions, movements, heart rates (HR), or respiration (Palumbo et al., 2017). Various social interactions—ranging from watching TV shows together to psychotherapy—are associated with synchrony (Cheong et al., 2023; Prinz et al., 2021), which is thought to enhance social bonds. Of note, however, synchrony may not be beneficial in all contexts (Wilson et al., 2018), and the ability to dynamically move in and out of synchrony may be crucial for healthy relating (Mayo & Gordon, 2020).

Synchronization predicts interpersonal processes such as attraction (Prochazkova et al., 2022). People who mirror each other’s movements, which is a form of synchrony, like each other more (Chartrand & Bargh, 1999). Moreover, inducing synchronous movement promotes feelings of closeness (Sharon‑David et al., 2018). In the context of dyadic meditations, the fostering of interpersonal synchrony could be a crucial mechanism explaining their efficacy. Järvelä et al. (2021) demonstrated that biofeedback on synchronized breathing and brain activity during dyadic meditation increased empathy and social presence, suggesting a mechanistic role for synchrony in dyadic meditations.

To build on previous findings, we tested a previously uninvestigated dyadic meditation, termed the *Just-Like-Me* (JLM) meditation. Here, participants silently gaze at each other while contemplating prompts that underscore shared human feelings, such as “just like me, this person has felt sadness, loneliness, and pain.” The aim is to cultivate a recognition of shared humanity, reminding individuals that certain feelings, needs, and wishes are universal (Dalai Lama et al., 2016). The exercise is described in Dass and Bush (2018). However, it has existed for longer, as it has been used since at least 2002 (M. De Wilde, personal communication, August 14, 2024), for example by teachers of non-violent communication (Rosenberg, 2003). No written documentation of the exercise prior to 2018 could be identified.

We compared JLM to an active control condition, namely mutual gazing (hereafter “Gazing”). The only difference from JLM was the lack of contemplation of prompts. Gazing can be considered one of the simplest dyadic meditations, and we predicted it would have positive impacts: 2 min of eye contact with a stranger induces affection (Kellerman et al., 1989). Thus, this design aimed to determine the specific effects of JLM, beyond those of Gazing alone. In Study 2, we also added a non-dyadic control condition, namely solitary meditation, to reveal effects of dyads beyond those of meditation per se. An advantage of both JLM and Gazing is that they can be easily integrated into daily life, meditation groups, or other contexts. They can be conducted in a brief format (here: 2 min per interaction), require minimal instruction or preparation, and can be adapted for both in-person and virtual contexts. Their simplicity also made them highly suitable for investigating the role of synchrony in dyadic meditations, as verbal interactions in other dyadic meditations can complicate the synchrony assessment. We thus test the effectiveness of JLM relative to Gazing on closeness and other beneficial social outcomes, and we assess the role of interpersonal synchrony in these dyadic meditations.

We examined the practices in virtual settings via a teleconferencing application (Study 1), and in-person (Study 2), as the COVID-19 pandemic spurred a widespread shift to remote work and virtual interactions (Gifford, 2022)—a change likely to persist in today’s world (Smite et al., 2023). The CovSocial project responded to this shift by studying app-mediated, audio-only dyads on a large scale. In the ReSource project, which took place before the pandemic, participants learned the dyads in-person before moving to virtual audio-only practice. Building on this foundation, the current study examined virtual, video-mediated dyads in Study 1 and fully in-person dyads in Study 2. By exploring both formats, our study provides further insights into fostering social connection in an increasingly digital world, where meaningful interactions are needed across both remote and in-person settings.

Our key psychological outcome was *interpersonal closeness*, defined as the degree to which participants feel connected (Kok & Singer, 2017). We also assessed *partner perceptions* (warmth, competence, attractiveness, potential for friendship) and *prosocial behavior*. Warmth relates to traits like kindness and trustworthiness (Cuddy et al., 2008). Competence—how capable and knowledgeable a partner appears—matters in contexts like the workplace. Attractiveness—being perceived as physically appealing—could be a desirable outcome of dyads in romantic contexts but a potentially problematic one at work. The perception of being suitable as a friend is relevant in various contexts. Prosocial behavior—defined here as behavior that benefits another (Pfattheicher et al., 2022; Schroeder & Graziano, 2015)—was measured using a hypothetical version of the dictator game, originally designed to assess fairness-related behavior (Kahneman et al., 1986). Here, participants allocated a fixed, hypothetical sum of money between themselves and another person, who was not informed of their decision. Finally, we assessed participants’ initial *motivation*, post-exercise *enjoyment*, and immediate *emotional response* to the meditation exercises.

Two types of synchrony were investigated. In Study 1, conducted virtually, we examined *smiling synchrony*—the coordinated expression of smiles between individuals (Mauersberger & Hess, 2019; Weber & Quiring, 2017). In Study 2, conducted in-person, we focused on *HR synchrony*. Among the myriad of facial expressions, smiling stands out as a potent social signal of positivity, cooperation, and approachability (Hess & Bourgeois, 2010), but it can also signal politeness or embarrassment (Niewiadomski et al., 2010). Synchronized smiling may have particularly positive effects on perceived empathy and interaction quality (Kim et al., 2009). HR synchrony, as well, may be a key correlate of connection, with studies in various settings revealing a role in interpersonal dynamics and co-regulation (Behrens et al., 2020; Coutinho et al., 2021; Flory et al., 2023).

Importantly, interpersonal synchrony may arise for many reasons and does not necessarily imply that an interpersonal interaction *caused* it (Palumbo et al., 2017). For example, individuals may respond similarly to a task or environment, or apparent synchrony may result from inherent, predictable patterns in the data (Denk et al., 2024). To address these possibilities, one can compare synchrony in actual, interacting pairs with that in *pseudo pairs*—pairs of individuals who were exposed to the same task but did not interact (Shiraishi & Shimada, 2021; Toppi et al., 2016). If synchrony is higher in actual pairs, it suggests that interpersonal influences—such as non-verbal interactions—played a role. This logic assumes that the environment and task are virtually identical across dyads.

We hypothesized that both JLM and Gazing amplify closeness, perceived warmth of the meditation partner, and monetary sharing in the dictator game. We further predicted that JLM would have stronger effects than Gazing, because JLM prompts add elements of perspective-taking and care. Moreover, we predicted both exercises would increase attractiveness ratings, as indicated by previous research (Shimojo et al., 2003). We did not predict any effect on competence ratings. Mechanistically, we hypothesized that people become closer by attending to each other and then synchronizing their behaviors and emotional arousal (Eilam, 2019; Nummenmaa et al., 2012). We predicted smiling synchrony and HR synchrony between meditation partners in JLM and Gazing, above and beyond synchrony found in pseudo pairs. Finally, we expected that smiling synchrony and HR synchrony would predict the degree to which dyad members feel closer to each other after the meditation, rate each other favorably, and share resources with each other (Kim et al., 2009).

## Study 1

### Method

#### Design

**Fig. 1:**
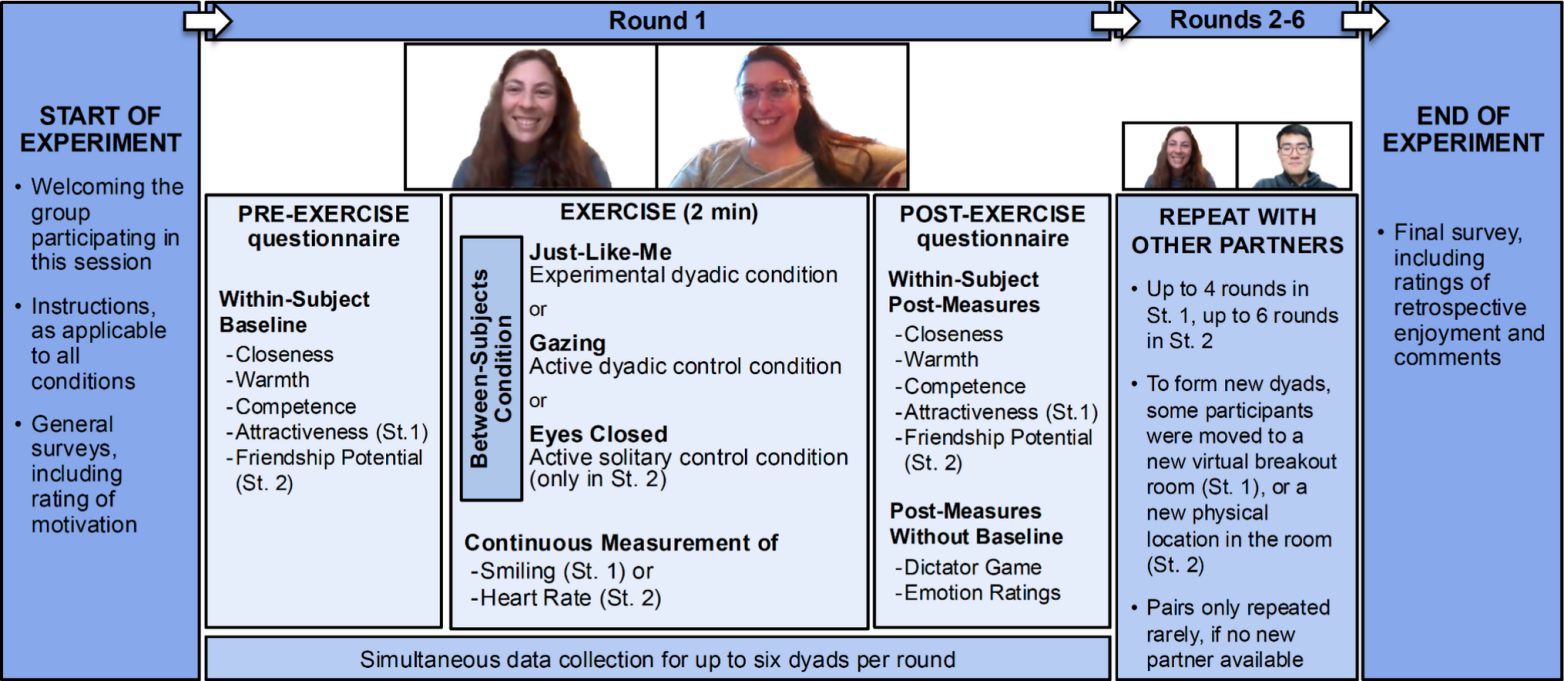
Experimental set-up. This procedure allowed us to collect data from up to 6 dyads in parallel and up to 6 rounds in series within approximately 1 hr. St., study

There were two conditions: JLM and Gazing. This dyadic design paired each participant serially with multiple others, measuring the same variables for both dyad members (Fig. 1). The approach was double-blind as far as practically feasible, as detailed in the following.

#### Procedure

Study 1 was conducted over 2 days in June 2021, in seven virtual group sessions with up to 11 participants each. We used the teleconferencing software *Zoom*. All participants in a session were assigned to the same condition. Assignment was not random, but participants chose their time slot without knowing which condition it corresponded to, introducing an element of randomness. Conditions were evenly distributed to control for time-of-day effects.

In the main Zoom room, participants were greeted by the lead experimenter (i.e., the lead author). They were asked to position themselves in front of the camera, ensure good lighting, and place their device on a stable surface. They were instructed to use “speaker view” and hide non-video participants to see their partner in full-screen. Each participant’s Zoom name was changed to an identifier letter (e.g., “Participant B”). Participants were then informed they would partake in brief meditative exercises with various partners and that they would receive the detailed instructions for these exercises later on (see full script and instructions in Online Resource 1). They were also informed that each 2-min meditation would be preceded and followed by a questionnaire. Participants were advised to engage naturally with the exercise, allowing for spontaneous reactions (e.g., smiling) if they happened but refraining from speaking.

We used a “speed dating”-like format, without any dating implications, with individuals sequentially paired with different partners. Participants were sent into Zoom breakout rooms in pairs. Here, assistants—not shown on camera—greeted them and asked them to complete the pre-exercise ratings in Qualtrics. After these ratings, meditation instructions (either for Gazing or JLM) were shown on screen for participants only. When both partners indicated they were ready, the meditation started. Participants were video-recorded side-by-side. Post-exercise, participants completed another survey. Following each round, they were re-paired for the next round and assigned to breakout rooms accordingly. Pairs were determined by the experimenter (e.g., A and B were paired in round 1, A and C in round 2). Whenever possible, participants were matched with new partners. For groups with odd numbers, one person per round took a break.

Blinding was applied as far as feasible: The experimenters were not aware which session corresponded to which condition. This was enabled because conditions were assigned to sessions by external staff. Moreover, the instructions for the exercise (JLM vs. Gazing) were shown in written text in the Qualtrics survey only, which was visible only to participants. Participants’ questions could have revealed the condition to assistants, but typically none were asked. Participants, in turn, did not know about the existence of the respective other condition and had no way of knowing if their condition was experimental or active control.

#### Conditions

##### JLM

Participants were instructed that they “will now look at each other for 2 min” and to “simply look at the other person’s face while contemplating the following sentence.” One of the following prompts was displayed underneath this text. Participants read the prompt, likely memorizing it, and then started Gazing: “Just like me, this person has felt sadness, loneliness and pain” (Round 1), “Just like me, this person has felt joy, fulfillment and gratitude” (Round 2), “Just like me, this person wishes to meet their needs and contribute to the needs of others” (Round 3), and “Just like me, this person longs for peace, love and self-expression” (Round 4).

##### Gazing

Participants were instructed to “simply look at the other person's face or eyes for 2 min” (Online Resource 1). We included the face as a target for viewing because it is difficult to hold eye contact on a teleconferencing application.

#### Measures

##### Closeness (Before and After Each Exercise)

The Inclusion of the Other in the Self Scale [IOS] (Aron et al., 1992) consists of seven pairs of circles labeled “self” and “other” varying in overlap (7-point). Participants chose “the image that best describes how close you currently feel with the person who is with you in the breakout room.” The scale has demonstrated high convergent, discriminant, and predictive validity, as well as reliability (Gächter et al., 2015).

##### Warmth, Competence, and Attractiveness (Before and After Each Exercise)

Participants rated their meditation partner on perceived warmth, “general competence in a work setting,” and attractiveness on a slider from 0 (*not at all*/*none*) to 100 (*completely*/*maximal amount*). Similar single-item measures have been used before (Fiske et al., 1999; Ludwig et al., 2022). Despite their limitations, they were chosen to avoid participant fatigue (Fiske et al., 2002).

##### Dictator Game (After Each Exercise)

To assess prosocial behavior, we used the dictator game, which has been widely used and validated (Engel, 2011; Franzen & Pointner, 2013; Kahneman et al., 1986). Participants were asked to hypothetically divide US$100 between themselves and their partner, without discussion (Online Resource 2). The slider ranged from 0 (*give all to partner*) to 100 (*keep all*).

##### Emotions (After Each Exercise)

Participants rated to what extent they felt anxious, relaxed, comfortable, and happy during the exercise on a slider from 0 (*not at all*) to 100 (*completely*). Such visual analogue scales are generally considered simple, valid, and reliable tools to assess subjective experience (Hasson & Arnetz, 2005; McCormack et al., 1988).

##### Motivation and Enjoyment

Once, in the beginning, participants indicated how motivated they were to try the experiment, and once, at the end, they indicated how much they had enjoyed it (four response options ranging from *Not at all* to *Very much*, see Online Resource 2).

##### Demographics, Meditation Experience, Pre-acquaintance, and Comments

Participants reported their age, gender, location, race, ethnic background, relationship status, student status, frequency of meditating, and experience with meditating with others (Online Resource 2). For each partner, participants also rated how well they knew them prior to the study (*extremely well*, *moderately*, or *not at all*). At the end, we asked participants whether anything was unclear during the experiment, how their experience was, and whether they had any suggestions.

##### Smiling

We captured facial expressions with the Zoom video recording functionality.

##### Other Measures Not Analyzed Here

There were a few additional questionnaires that were part of a different research project and outside the scope of this paper (Online Resource 2).

##### Attention Checks

Attention checks were included throughout the surveys (Online Resource 3).

#### Participant and Dyad Characteristics

Fifty-five participants were recruited from an existing data bank via the Wharton Behavioral Lab at the University of Pennsylvania (78% full-time students; Gazing, 28; JLM, 27). Responses from one further participant were missing, but their partner’s responses were included. Most participants resided in the USA (one participant in Canada). The majority (88%) had never meditated together with others; 12% said they had. More characteristics are shown in Table 1.

**Table 1:** Sample characteristics for Study 1

Gender	Age	Race and ethnicity	Relationship status	Meditation frequency
39 female, 16 male	*M* = 23.55, *SD* = 7.4	42% Asian, 33% White, 18% Black or African American, 2% American Indian or Alaska Native, 6% mixed race or other; 15% Spanish, Hispanic, and/or Latino	60% single/not dating, 9% casually dating, 29% in committed relationships (unmarried), 2% married	53% never/almost never, 31% less than once a month, 13% a few times per month, 4% a few times per week, 0% daily

This resulted in 101 dyads (Gazing, 49; JLM, 52) with normally two survey response sets per dyad (one per dyad member). Two of the dyads each had one response set missing, resulting in 200 response sets in total (Gazing, 98, JLM, 102). Thirty-eight percent of the dyads were mixed-gender, and 62% were same-gender dyads. Ninety-six were unique pairings, and five pairings were repeated. For most dyads, partners indicated that they did not know each other at all. In two dyads, both partners knew each other *moderately* well. In three dyads, familiarity ratings were mixed (*moderately* vs. *not at all*; two of these mixed ratings were from repeat pairs).

#### Power Analysis

We conducted a post hoc power analysis to estimate the minimum detectable effect sizes for both within- and between-condition comparisons, adjusting for repeated measures and participant clustering, using the package *pwr* in R (Champely et al., 2018). We set the desired power to 0.80 and the significance level to *α* = 0.05 (see Online Resource 4 for details). This analysis was focused on the key variable closeness.

#### Data Analyses

All analyses were carried out using R version 4.1.3 (R Core Team, 2021). We used chi-square tests and independent *t*-tests to assess differences between groups, using the *compareGroups* package in R (Subirana et al., 2014). For motivation and enjoyment, we used Wilcoxon rank sum tests to compare JLM and Gazing. We also conducted Spearman rank-order correlations to assess the link between motivation and enjoyment ratings.

To test for effects of the exercises, we carried out multilevel modeling for dyadic data (one model per variable), thus taking into account the interdependence of two dyad members (Atkins, 2005; Hoffman & Rovine, 2007; Kenny et al., 2006). We used the R package *lmerTest* (Kuznetsova et al., 2017). Variables for which we had pre-exercise values were standardized relative to their Pre-Exercise *Mean* and *SD*. The other variables were *z*-scored.

For closeness, warmth, competence, and attractiveness, fixed factors were pre-exercise values on each variable, condition (Gazing vs. JLM), and their interaction. For the models for the dictator game and emotions (anxiety, comfort, relaxation, happiness), the only fixed effect predictor was condition, as no pre-exercise values were available. To explore the link between facial expressions and self-reported affect, we also formulated models predicting the four emotions from mean smiling, condition, and their interaction. Condition was coded as a dummy variable with 1 for JLM and 0 for Gazing, thus setting Gazing as the reference condition in all Study 1 models. In regression models, the intercept denotes the effect when all predictors are set to 0. Here, the intercept thus denotes the effect of Gazing (condition JLM = 0) for a participant with average pre-exercise levels (*z*-score = 0, if pre-exercise values are included in the respective model). As condition was encoded with 1 for JLM, any effects of condition in the models denote JLM’s effects above and beyond (or below) those of Gazing. In each model, we initially included random intercepts for participant ID, dyad ID, partner ID, and round. However, including all four of them led to fitting issues. To address this, we removed the worst-performing random intercepts per model (explaining variance close to 0), while making sure this did not affect the results (details in Online Resource 5).

We extracted estimates of smiling behavior over the 2-min exercises using Affectiva AFFDEX 5.1 (Online Resource 6). Data were rounded to whole numbers and smoothed over a 160 ms window (four samples) to emphasize real expression changes and reduce noise, using the *zoo* package in R (Shen et al., 2012; Yan et al., 2013). As a measure of overall synchrony, we calculated Pearson’s correlations between these smiling values for dyad partners across the entire 2 min of the exercise (Parkinson et al., 2018). Despite several limitations (Palumbo et al., 2017), correlations are a widely used and straightforward indicator of synchrony (Chanel et al., 2012; Ebisch et al., 2012; Schneider et al., 2020). To ensure that non-normality of the data did not cause issues, we also repeated the analysis using Spearman’s correlations. The results were comparable. However, in line with others, we report Pearson’s correlations here, as they reflect the actual intensity of smiling, rather than just the relative similarity in smiling behavior. To establish a strict comparative baseline, we artificially paired each participant’s data with the data of all other possible partners of the same condition that were not actually their partners (*pseudo pairs*) and calculated the smiling correlation for each (Flory et al., 2023).

We then formulated a linear regression model predicting smiling correlation from condition, type (real pair vs. pseudo pairs), average smiling, and their interactions, on the dyad level. The reference for condition in the model was set to Gazing, and the reference for type was set to pseudo pairs. Next, we assessed whether smiling behavior during exercises predicted changes in partner ratings for the real pairs. To do so, we formulated linear models with the predictor variables smiling correlation, average smiling per dyad, condition, and their interactions, again on the dyad level. The outcome variable was the dyad-specific change of each of the variables (post-values minus pre-values, averaged across the two dyad members), in separate models for closeness, warmth, competence, and attractiveness. We also estimated a model with the outcome dictator game allocations, averaged across the two dyad members. As a sanity check, we repeated the models for pseudo pairs. Variables were *z*-scored. We also conducted supplemental analyses on the effects of gender (mixed-gender vs. same-gender dyads). There were few gender-related effects, and for conciseness, these are reported in Online Resource 7.

To further explore the data, we also visualized synchrony across time using a windowed cross-lagged correlation analysis (Riehle et al., 2017). This approach also accounts for potential delays in one person’s smile response to the other’s while allowing shifts in leader–follower dynamics over time (e.g., sometimes A smiles first and B follows, and at other times, vice versa). The method involves a sliding window of fixed size moving along the timeline in increments (Boker et al., 2002). Within each window, various time lags (i.e., shifts in one data set relative to the other) are introduced before calculating correlations. We computed cross-correlations using the ccf() function in R, applying a window of 60 s (1500 samples per window) which moved across the data in 1-s (25-sample) increments. This large window size was chosen to accommodate the sparse and variable nature of smiles across different periods and participants. For each window, we calculated correlations across various lags. Given evidence that most positive facial reactions to others occur within ± 1 s (Cheong et al., 2023; Riehle et al., 2017), we permitted a lag of ± 1 s in responses to a partner’s smiling. The lag was applied in steps of 0.04 s (1 sample). For each window, we recorded the maximum positive correlation across all lags to quantify peak synchrony within that interval, irrespective of which partner initiated the smile or the lag length. We then plotted mean peak synchrony across time for real pairs and pseudo pairs, with 95% confidence intervals (*CI*). As a final robustness check, we repeated all key analyses (partner ratings, dictator game, and synchrony analyses) after excluding individuals who reported feeling familiar with each other (Online Resource 8).

### Results

#### Attention Checks and Allocation to Groups

All participants passed attention checks (Online Resource 3). Groups did not differ on gender, age, race, relationship status, or meditation experience (*p*-values > 0.08, Online Resource 9).

#### Achieved Power

Regarding our key outcome measure pre-to-post changes in closeness, the study had 80% power to detect minimum effect sizes of *d* = 0.38 for JLM and *d* = 0.42 for Gazing (small: *d* = 0.2, medium: *d* = 0.5, large: *d* = 0.8; Cohen, 1988). For between-group comparisons, the estimated minimum detectable effect size was *d* = 0.56. Thus, the study was well-powered to detect within-subject effects but was only powered to detect moderate-to-large differences between conditions. Smaller group differences may be missed.

#### Motivation and Enjoyment

Most participants were not (50.9%) or just somewhat (38.2%) motivated to try the exercises (10.9% not at all). Yet, afterwards, the majority said they enjoyed it at least a little bit (7.3% very much, 30.9% moderately, 45.5% a little bit, and 16.4% not at all). Participants’ motivation (*W* = 370, *p* = 0.88) and enjoyment (*W* = 474, *p* = 0.08) did not differ between conditions. Motivation and enjoyment were positively correlated (Spearman’s* r* = 0.47, *p* < 0.001).

#### Effects on Closeness, Warmth, Competence, and Attractiveness

**Fig. 2:**
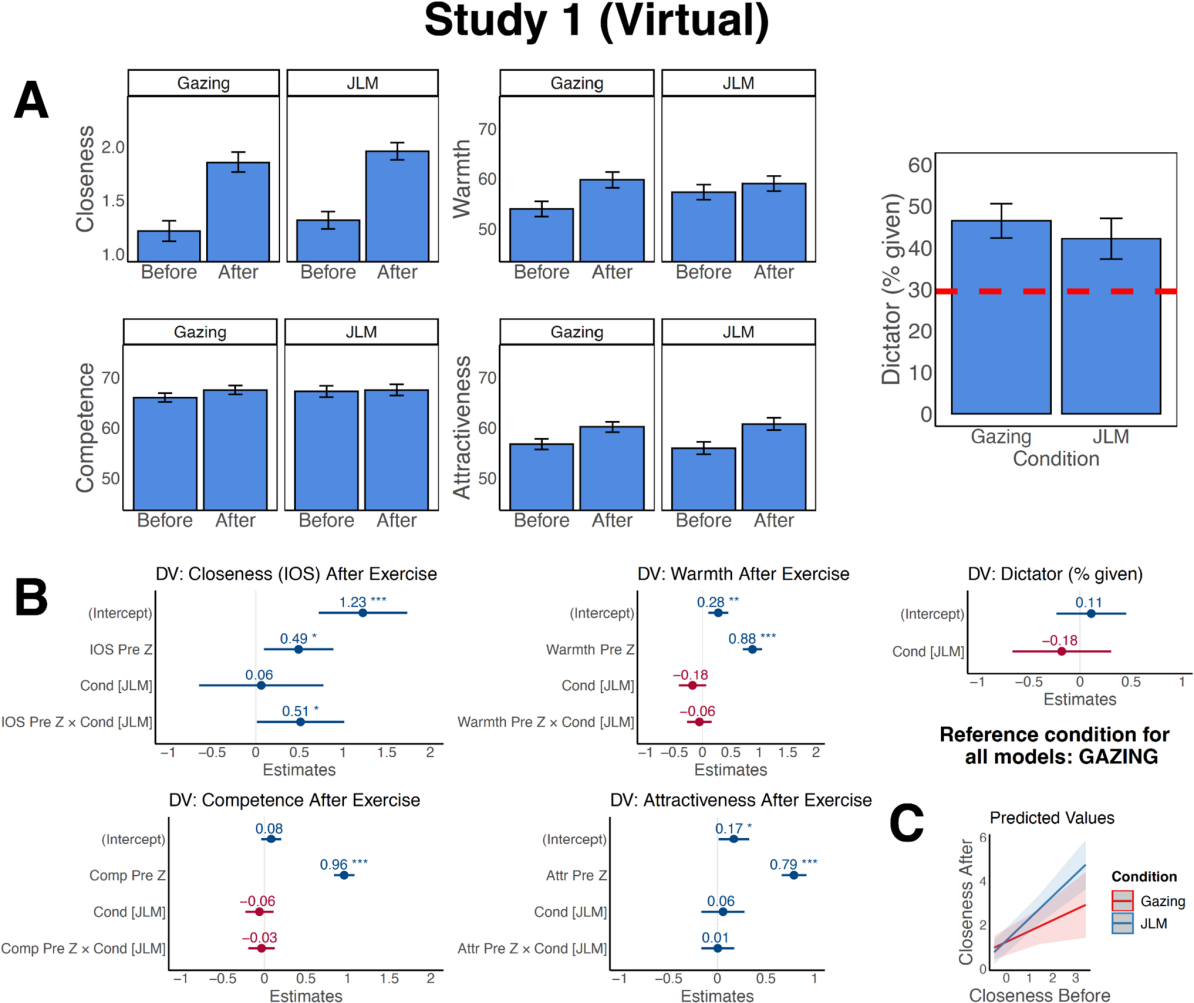
Virtual Gazing and JLM exercises were associated with increased closeness, perceived attractiveness, and prosocial behavior. **A** Average pre-exercise and post-exercise values for closeness, warmth, competence, and attractiveness for both conditions, and results for the dictator game. Plots are based on individual ratings by participants per dyad. Gazing *n* = 98, JLM *n* = 102. The dashed red line in the dictator game plot indicates the average proportion shared reported in a recent meta-study (Doñate‑Buendía et al., 2022) for comparison. Error bars denote 95% *CI*, adjusted for within-subject designs where appropriate (Loftus & Masson, 1994). Note that for simplification and visualization, this figure treats each repeat participation of the same individual as independent and treats each participant as independent of their dyad partner. For appropriate statistical testing that takes into account these interdependencies, refer to panel B. **B** Results from the mixed-effects model. Values in the figure are the standardized beta values. *p < 0.05, **p < 0.01, ***p < 0.001. Gazing was the reference category. **C** Visualization of the interaction effect of pre-exercise closeness with condition on post-exercise closeness. Predicted values based on the model

Gazing significantly increased closeness (*β* = 1.23, *SE* = 0.26, *p* < 0.001), warmth (*β* = 0.28, *SE* = 0.09, *p* = 0.008), and perceived attractiveness (*β* = 0.17, *SE* = 0.08, *p* = 0.037), as indicated by significant intercept terms (as the reference condition was Gazing; Fig. 2A and B). There was no effect on competence (*β* = 0.08, *SE* = 0.06, *p* = 0.19). These standardized coefficients indicate that participants felt 1.23 *SD* closer to their partner after Gazing compared to before. JLM added an additional 0.06 *SD*, but the difference between Gazing and JLM was not significant (condition JLM: *β* = 0.06, *SE* = 0.36, *p* = 0.86). There were also no significant differences between conditions for any of the other variables (Warmth: *β* = − 0.18, *SE* = 0.12, *p* = 0.15; Attractiveness: *β* = 0.06, *SE* = 0.11, *p* = 0.60; Competence: *β* = − 0.06, *SE* = 0.09, *p* = 0.49). Since the effect of JLM was *negative* (relative to Gazing) for warmth, we explored changing the reference condition of the model from Gazing to JLM. The intercept was not significantly different from 0, indicating that JLM did not increase perceived warmth. In sum, virtual Gazing increased closeness, warmth, and attractiveness, while JLM increased closeness and attractiveness, and there were no significant differences between conditions on these variables.

There was an effect of pre-exercise ratings for all variables (e.g., Closeness: *β* = 0.49, *SE* = 0.20, *p* = 0.02). That is, initial feelings of closeness, warmth, attractiveness, and competence predicted these perceptions after the exercise. There was an interaction effect of pre-exercise closeness ratings with condition (*β* = 0.51, *SE* = 0.25, *p* = 0.04), but otherwise there were no interaction effects. The interaction was driven by individuals with higher pre-exercise closeness reporting relatively greater increases in closeness for JLM compared to Gazing, whereas for those with lower pre-exercise closeness, the type of exercise mattered less (Fig. 2C; but see Online Resource 8 for evidence suggesting a potential confounding role of familiarity in this interaction).

#### Effects on Dictator Game

There was no effect of condition on dictator game allocations (*β* = − 0.18, *SE* = 0.24, *p* = 0.47; Fig. 2B). To explore whether this was due to neither exercise increasing sharing or both having equal effects, we compared our results to the average dictator game sharing of 29.6% found in a meta-analysis of 136 studies, across various settings (Doñate‑Buendía et al., 2022). Allocations in both conditions were numerically higher (JLM 42.2%, *SD* = 24.8; Gazing 46.4%, *SD* = 20.5).

#### Effects on Emotions

**Fig. 3:**
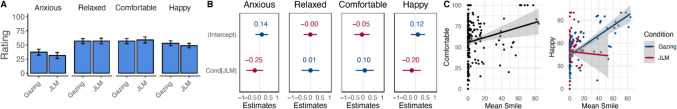
Self-reported emotions after the virtual dyads. **A** Average emotions. Plots are based on individual ratings per dyad. For visualization, this figure treats each data set as independent. For appropriate statistical testing, refer to panel B. **B** Results from the mixed-effects model. Values are the standardized beta values. Gazing was set as the reference condition.** C** Mean smiling and emotion ratings. Each dot corresponds to one participant in one dyad. For visualization, this plot and the fit lines treat each data point as independent. For statistical testing, refer to the text. *Note.* Gazing *n* = 98, JLM *n* = 102. Error bars denote 95% *CI*

Participants reported moderate comfort, relaxation, and happiness (Fig. 3A), and relatively low anxiety (*M* across conditions: 34.18 out of 100, *SD* = 27.42), with no differences between conditions (*p*-values > 0.29, Fig. 3B). There were also no gender composition effects.

#### Smiling Results

##### Smiling Predicted Self-reported Affect

Mean smiling during the meditations predicted subsequent comfort (*β* = 0.20, *SE* = 0.08, *p* = 0.015) and happiness ratings (*β* = 0.50, *SE* = 0.08, *p* < 0.001), after controlling for condition. For happiness, this effect was stronger for Gazing (interaction Gazing × average smiling *β* = − 0.39, *SE* = 0.18, *p* = 0.028, Fig. 3C). No other main effects or interactions were significant (all *p*-values > 0.20).

##### Smiling Across Conditions

**Fig. 4:**
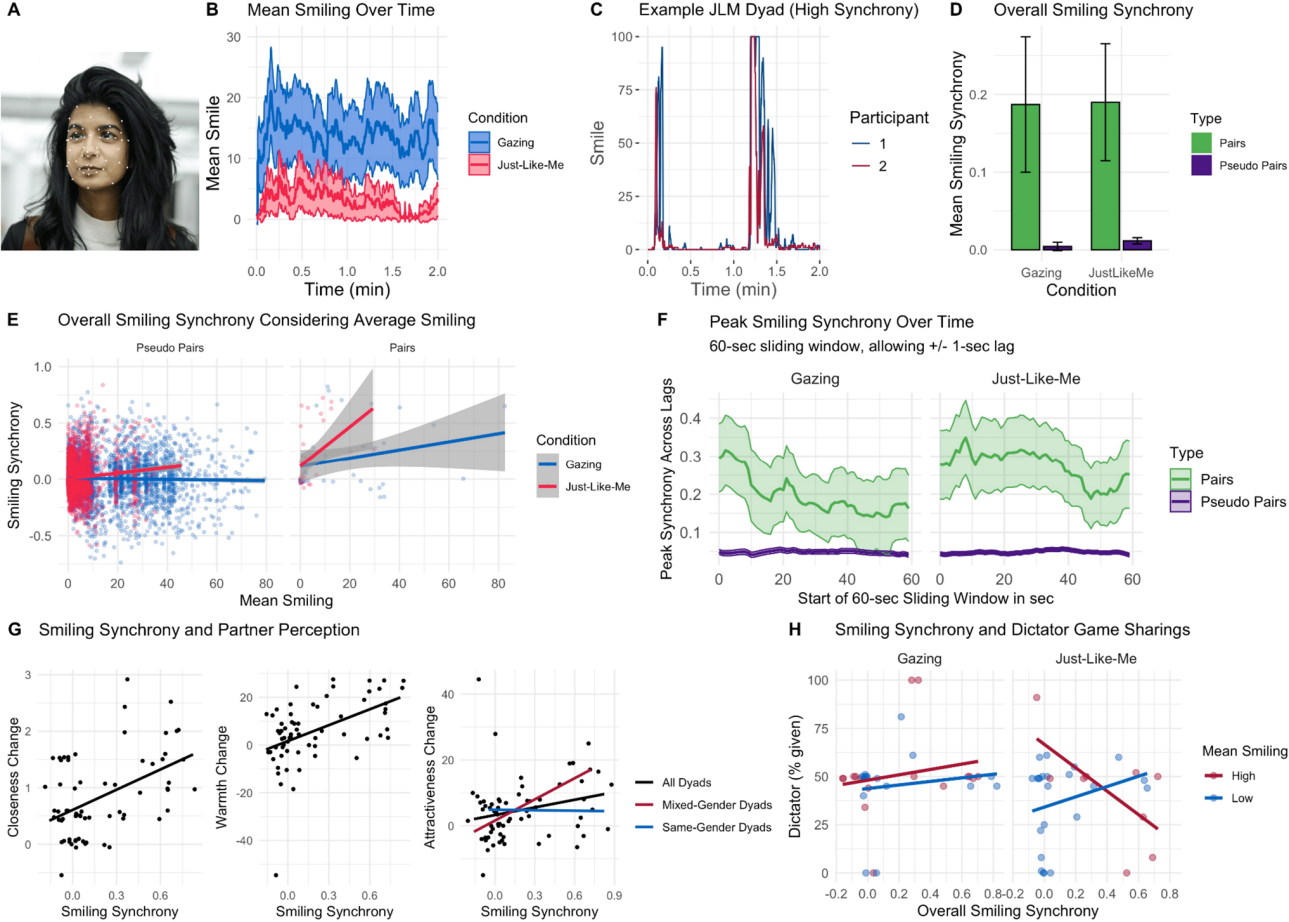
Smiling synchrony was present in both conditions and predicted increases in closeness, warmth, and attractiveness.** A** Facial muscle tracking with Affectiva Software: Affectiva estimated emotional expressions by assessing facial muscle movements from Zoom recordings, allowing for the extraction of smiling measures. Image courtesy of iMotions (2024). **B** Mean smiling intensity across time for the two conditions. **C** Example time course from a JLM dyad with high smiling synchrony. **D** Overall smiling synchrony across conditions. This measure refers to the overall Pearson’s correlation of the full two time series of each dyad. **E** Link between average smiling and synchrony across conditions for real and pseudo pairs. Linear trend lines fitted per condition.** F** Time course of peak synchrony while allowing for a lag between responses. The *X*-axis refers to the beginning of the sliding window, up to 2 min in total. **G** Links between smiling synchrony and partner perceptions. Smiling synchrony predicted increases in perceived closeness, warmth, and, for mixed-gender dyads, attractiveness. For simplification and visualization, regression lines result from a simple model predicting changes in the variables from smiling synchrony alone, based on original (not *z*-scored) values and separated by mixed-gender vs. same-gender dyads for attractiveness. Given these effects did not differ across conditions, this graph does not distinguish conditions. Change scores refer to mean change across both dyad members. **H** Links between smiling synchrony and dictator game sharing. Participants’ smiling levels were categorized as “Low” or “High” (i.e., below or above the condition-specific mean). Linear trend lines are shown per group. Note. Each dot represents one dyad. Plots do not take into account repeat participation of participants and treat each dyadic interaction independently. All shaded ribbons and error bars indicate 95% CI of the mean

Gazing participants smiled more overall (*M* = 13.7, *SD* = 29.8) compared to JLM participants (*M* = 2.89, *SD* = 13.8; Fig. 4A and B). Given this finding, it was imperative to control for average smiling in our models below.

##### Overall Smiling Synchrony Across Conditions

Smiling synchrony could be assessed in 65.3% of dyads (*n* = 66), where both participants smiled at some point during the meditation (69.4% of Gazing dyads and 61.5% of JLM dyads). For the remaining dyads, at least one participant did not smile at all, so smiling synchrony could not be evaluated. These dyads were therefore excluded from this analysis.

Among the included dyads, real pairs in both conditions exhibited smiling synchrony significantly greater than zero (Fig. 4C–D). Synchrony for JLM was higher than for Gazing after controlling for average smiling (Fig. 4E; JLM *β* = 0.13, *SE* = 0.03, *p* < 0.001). Moreover, pairs showed higher synchrony than pseudo pairs (*β* = 0.86, *SE* = 0.19, *p* < 0.001). The main effect of average smiling was not significant (*β* = − 0.02, *SE* = 0.02, *p* = 0.15), but several interactions emerged. There was an interaction between condition JLM and dyad type (*β* = 0.70, *SE* = 0.31, *p* = 0.02), suggesting that the effect of JLM on synchrony was stronger for pairs than for pseudo pairs. Furthermore, there was an interaction between JLM and average smiling (*β* = 0.23, *SE* = 0.05, *p* < 0.001), indicating that higher average smiling was associated with greater synchrony for JLM. An interaction between type and smiling also emerged (*β* = 0.31, *SE* = 0.12, *p* = 0.009), with average smiling enhancing synchrony more strongly for pairs than for pseudo pairs. Finally, there was a three-way interaction between condition, dyad type, and average smiling (*β* = 0.88, *SE* = 0.39, *p* = 0.025), indicating that synchrony for JLM was particularly enhanced for real pairs with high smiling. Figure 4F shows the time course of peak smiling synchrony for both conditions and dyad types.

##### Smiling Synchrony Predicted Partner Perceptions

Greater synchrony was associated with stronger increases in closeness (*β* = 0.56, *SE* = 0.19, *p* = 0.004) and warmth (*β* = 0.55, *SE* = 0.19, *p* = 0.006), after controlling for average smiling and condition. This was not the case for competence (*β* = 0.07, *SE* = 0.17, *p* = 0.70) or attractiveness (*β* = 0.25, *SE* = 0.20, *p* = 0.23). A supplemental analysis (Online Resource 7), however, revealed an effect of gender composition in the attractiveness analysis: In mixed dyads—but not in same-gender dyads—smiling synchrony was linked with stronger increases in attractiveness (Fig. 4G, synchrony × mixed-gender *β* = 0.62, *SE* = 0.27,* p* = 0.03). Average smiling did not impact any outcomes (all *p*-values > 0.10). Moreover, in pseudo pairs, there were no effects of synchrony on partner perceptions (all *p*-values > 0.05).

##### Smiling Behavior Predicted Dictator Game Sharing

Synchrony did not have a main effect on sharing (*β* = 0.18, *SE* = 0.19, *p* = 0.36), and neither did average smiling (*β* = 0.10, *SE* = 0.13, *p* = 0.46). However, there was an interaction of JLM with synchrony (*β* = − 0.86, *SE* = 0.39, *p* = 0.03), and a three-way interaction of synchrony, average smiling, and JLM (*β* = − 1.67, *SE* = 0.75, *p* = 0.03), both with large effect sizes. For JLM participants with high average smiling, higher synchrony predicted lower sharing (Fig. 4H), while for JLM participants with low average smiling and for Gazing, there was no or a slightly positive relationship between synchrony and sharing. In pseudo pairs, there was an interaction of synchrony with average smiling with a very small effect size (*β* = − 0.06, *SE* = 0.03, *p* = 0.03), but no other effects involving smiling synchrony.

#### Robustness Analysis: Exclusion of Familiar Dyads

Our results for partner ratings, the dictator game, and synchrony remained robust when analyzing only unfamiliar participants (Online Resource 8). The only exception was the interaction between pre-exercise closeness and JLM on post-exercise closeness.

#### Textual Responses

Comments can be found in Online Resource 11. Feedback varied, describing the experiment as interesting, enjoyable, uncomfortable, or anxiety-inducing. The virtual format, particularly the difficulty of connecting via video, was frequently cited as a drawback. Many felt initial awkwardness that decreased over time, and some felt self-conscious about attractiveness ratings.

## Study 2

### Method

#### Design

Study 2 implemented the exercises in an in-person context. The study mirrored Study 1’s design but with three conditions: JLM, Gazing, and “Eyes Closed.” The latter was a non-dyadic control condition in which people were sitting next to each other with their eyes closed, observing their own breath. The attractiveness rating before and after each exercise was removed because several Study 1 participants reported discomfort with it, possibly impacting the exercises. Instead, we included a rating of the potential for friendship in each round, and inquired about romantic-sexual attraction only at the end of the experiment, retrospectively.

#### Procedure

The experiment spanned 12 in-person group sessions over 4 days in April 2022. Upon arrival, participants put on an HR belt and a letter tag for anonymous identification (e.g., “B”). Participants were then seated in pairs at one of up to six stations with one computer per participant for survey completion in Qualtrics.

All sessions were again led by the lead author, sometimes supported by assistants. The lead experimenter greeted participants and gave a general introduction, as for Study 1 (Online Resource 1). Each round began with participants completing pre-exercise questions about their assigned partner on their computers. This was followed by on-screen instructions for the upcoming exercise. Once preparations were complete, the experimenter signaled the start of the exercise and simultaneously initiated HR recording for all participants. After approximately 2 min, the recording was stopped, and post-exercise questions were completed. Afterwards, half the participants rotated to new stations for a different pairing (e.g., Participant B moved from Participant A to Participant C). This process was repeated up to six times.

As in Study 1, the experimenter and assistants initially did not know the assigned condition for each session. Blinding was only fully effective at the start, as—in practice—experimenters could often deduce the condition during the session when observing participants (e.g., Eyes Closed looked different from Gazing). Participants were unaware of the other conditions tested in the study.

#### Conditions

The instructions for all conditions were shown on the screen, as follows (see Online Resource 1).

##### JLM

“Please look into the other person's eyes while contemplating the following sentence.” Prompts in rounds 1–4 matched those in Study 1. We added two more rounds: “Just like me, this person has felt unworthy or inadequate,” and “Just like me, this person wishes to be loved.”

##### Gazing

“Please look into the other person's eyes for 2 min.”

##### Eyes Closed

“In this exercise, we simply ask you to close your eyes and observe your own breath for 2 min.” Note that participants first moved next to the partner of the respective round, and so they were doing this exercise alongside the other person.

#### Measures

All measures were equivalent to Study 1 except for the following (see Online Resource 2 for details).

##### Friendship

The attractiveness rating before and after each exercise was replaced by “When I look at my current partner, I think there is potential for a real friendship.” (slider from 0 to 100).

##### Romantic Sexual Attraction

We retrospectively queried participants about attraction for each exercise partner, both pre- and post-exercise, at the end of the experiment.

##### Masks and Adherence

At the time of this study, pandemic-related mask mandates had just been lifted. Participants reported on their mask use and their adherence to exercise instructions.

##### HR

During the exercises, HR was measured in beats per minute (bpm) at 0.5 Hz using Polar chest belts (H10). All belts wirelessly transmitted data simultaneously to a single tablet using Polar Team software. This software uses a synchronized start and end time per recording across all participants. Our setup did not provide HR variability or waveform shape.

#### Participant and Dyad Characteristics

There were 98 participants (Gazing, 35; JLM, 33; Eyes Closed, 30; 92% students; Table 2 for more details), again recruited via the Wharton Behavioral Lab. Data from one further participant were excluded due to their serving as a research assistant in Study 1. Otherwise, none had participated in Study 1. Nineteen percent had experience meditating with others (e.g., in groups), 69% did not, and 11% did not respond. Seventeen percent wore a mask during the study, 3% did so for most of the session, 4% briefly, and 76% never wore one.

**Table 2:** Sample characteristics for Study 2

Gender	Age	Race and ethnicity	Relationship status	Meditation frequency
63 female, 35 male	*M* = 21.35, *SD* = 5.99	55% Asian, 32% White, 11% Black or African American, 2% mixed race or other; 6% Spanish, Hispanic, and/or Latino	53% single/not dating, 14% casually dating, 30% in a committed relationship (unmarried), 2% married, 1% missing	46% never or almost never, 32% < 1 × a month, 16% few times per month, 4% few times per week, 1% daily, 1% missing

Participants completed three to six rounds with different partners, resulting in 238 dyad pairings (JLM, 89 dyads; Gazing, 80 dyads; Eyes Closed, 69 dyads). Forty-three percent were mixed-gender dyads, and 57% were same-gender. Most were unique pairings; only for one dyad was the pairing repeated. In 221 dyads, both members were unfamiliar with each other; 7 dyads knew each other moderately well, 5 were extremely familiar, and 5 reported mixed familiarity (2 dyads: *extremely well* vs. *moderate*, 3: *not at all* vs. *moderate*). No dyads had maximum pre-exercise Closeness ratings, allowing room for increases even among well-acquainted pairs. Thus, no dyads were excluded based on prior familiarity. For HR, there were 468 complete data files (JLM, 178; Gazing, 160; Eyes Closed, 130). For two participants, HR data were missing across all rounds due to insufficient HR belts. In 231 out of 238 dyads, HR data were available for both participants, allowing their inclusion in the synchrony analysis.

### Power Analysis

An approximate post-hoc power analysis was conducted as for Study 1, for the main outcome variable closeness, with *α* = 0.05 and power = 0.80 (Online Resource 4 for details).

### Data Analyses

Most analyses were carried out as in Study 1, except for the following points. Self-reported adherence—not included in Study 1—was regressed on conditions JLM and Gazing. We used the Kruskal–Wallis test to assess differences in enjoyment and motivation across the three conditions.

To model conditions in all the mixed-effects models, we included a dummy variable for Gazing (1 for Gazing, 0 for JLM or Eyes Closed) and a dummy variable for JLM (1 for JLM, 0 for Gazing or Eyes Closed). Thus, Eyes Closed was set as the reference condition in Study 2. Due to the novel model characteristics, the intercept in these models denotes the effect of the Eyes Closed condition for participants with average pre-exercise values (i.e., when all model components except the intercept equal 0). The JLM dummy variable denotes the difference between JLM and Eyes Closed. This is different from Study 1, where the JLM variable denoted the difference between JLM and Gazing because Eyes Closed was not available as a reference condition. The Gazing dummy variable denotes the difference between Gazing and Eyes Closed. For the exploration of HR, we tested whether raw HR differed between the start and the end of the exercises using intercept-only mixed-effects models for each condition. The outcome variable was HR change (final minus first timepoint), with random intercepts included to account for repeated participation. For details on modeling, such as random intercepts, see Online Resource 5.

HR data, as a continuous physiological signal, exhibits inherent autocorrelation due to slow cyclic fluctuations such as breathing. This may artificially inflate synchrony between two time series (Dean & Dunsmuir, 2016; Denk et al., 2024). To address this, we applied an autoregressive (AR) model using the ar() function in R, thereby reducing autocorrelation and extracting the residuals (termed *prewhitening*). We then measured HR synchrony by calculating Pearson’s correlation coefficient between the pre-whitened HR of the two members of each dyad. Again, despite limitations (Palumbo et al., 2017), this is a straightforward and common approach to quantify whether two time series covary (Chanel et al., 2012; Ebisch et al., 2012; Schneider et al., 2020). The low temporal resolution of our data (0.5 Hz, i.e., one sample every 2 s) makes it less suitable for more complex analyses that capture finer temporal dynamics. In addition, we calculated HR correlations for *pseudo pairs*—participants in the same condition but not paired at that moment (Palumbo et al., 2017). We then formulated linear regression models predicting HR correlation from the predictors condition, type (pairs vs. pseudo pairs), average HR, and their interactions. However, due to the complexity of the model (three conditions, two dyad types, and interactions with average HR), various issues related to multicollinearity and interpretability of the coefficients emerged. Therefore, we opted to report a straightforward analysis: We conducted a series of independent *t*-tests to examine synchrony differences in pairs vs. pseudo pairs within each condition (JLM, Gazing, Eyes Closed) and between conditions among pairs (JLM vs. Gazing, Gazing vs. Eyes Closed, JLM vs. Eyes Closed), while applying Bonferroni correction for multiple comparisons. Note that the more complex modeling approaches, including those controlling for average HR, led to the exact same conclusions as the *t*-tests.

Next, we regressed the difference scores for our self-report outcomes (closeness, warmth, competence, and friendship potential after the exercise minus before) onto HR synchrony values for real pairs only. We also included condition and average HR per dyad in the model. Since no significant results were found, we did not repeat this analysis for pseudo pairs. All variables were *z*-scored. Finally, we again visualized synchrony across time approximately using WCLC (Boker et al., 2002). The window size was again set to 60 s, the step size was 2 s (one sample), and the maximum lag was set to 2 s (one sample). Finally, a robustness check was again conducted by excluding participants who were familiar with their meditation partner. Key analyses were repeated in the reduced sample for partner ratings (excluding attractiveness, as it was assessed retrospectively in Study 2), dictator game decisions, and HR synchrony.

### Results

#### Attention Checks, Allocation to Groups, and Adherence

All participants passed attention checks (Online Resource 3). The three groups did not differ on gender, age, race, meditation experience, or relationship status (*p*-values > 0.20, Online Resource 10). Participants reported adhering less well to JLM instructions (*M* = 82.64, *SD* = 19.89) compared to Gazing (*M* = 94.31, *SD* = 7.41) and Eyes Closed (*M* = 93.83, *SD* = 9.85; *β* JLM = − 0.78, *SE* = 0.24, *p* = 0.001). There were no differences between Gazing and Eyes Closed (*β* Gazing = 0.03, *SE* = 0.23, *p* = 0.89).

#### Achieved Power

Study 2 was well-powered to detect small-to-moderate effects for both within-condition effects and differences between conditions for the closeness outcome variable. That is, the minimum detectable effect sizes for within-subject effects were Cohen’s *d* = 0.36 for JLM, *d* = 0.33 for Gazing, and *d* = 0.36 for Eyes Closed. For group comparisons, the minimum detectable effect size was Cohen’s *f* = 0.22. Reference values are *d* = 0.2 or *f* = 0.10 for small, *d* = 0.5 or *f* = 0.25 for medium, and *d* = 0.8 or *f* = 0.40 for large effects (Cohen, 1988).

#### Motivation and Enjoyment

Most participants (65.3% across all conditions) were somewhat motivated to try the exercises, and 19.4% were very motivated. Only 14.3% reported no motivation at all. Afterwards, 13.3% said they enjoyed the experiment very much, 39.8% moderately, 35.7% a little bit, and 11.2% not at all. Motivation did not differ across conditions (Kruskal–Wallis *χ*^2^ = 2.99, *p* = 0.22), and neither did enjoyment (Kruskal–Wallis *χ*^2^ = 0.29, *p* = 0.86). Motivation and enjoyment were positively correlated, Spearman’s *r* = 0.44, *p* < 0.001.

#### Effects on Closeness, Warmth, Competence, Potential for Friendship, and Attraction

**Fig. 5:**
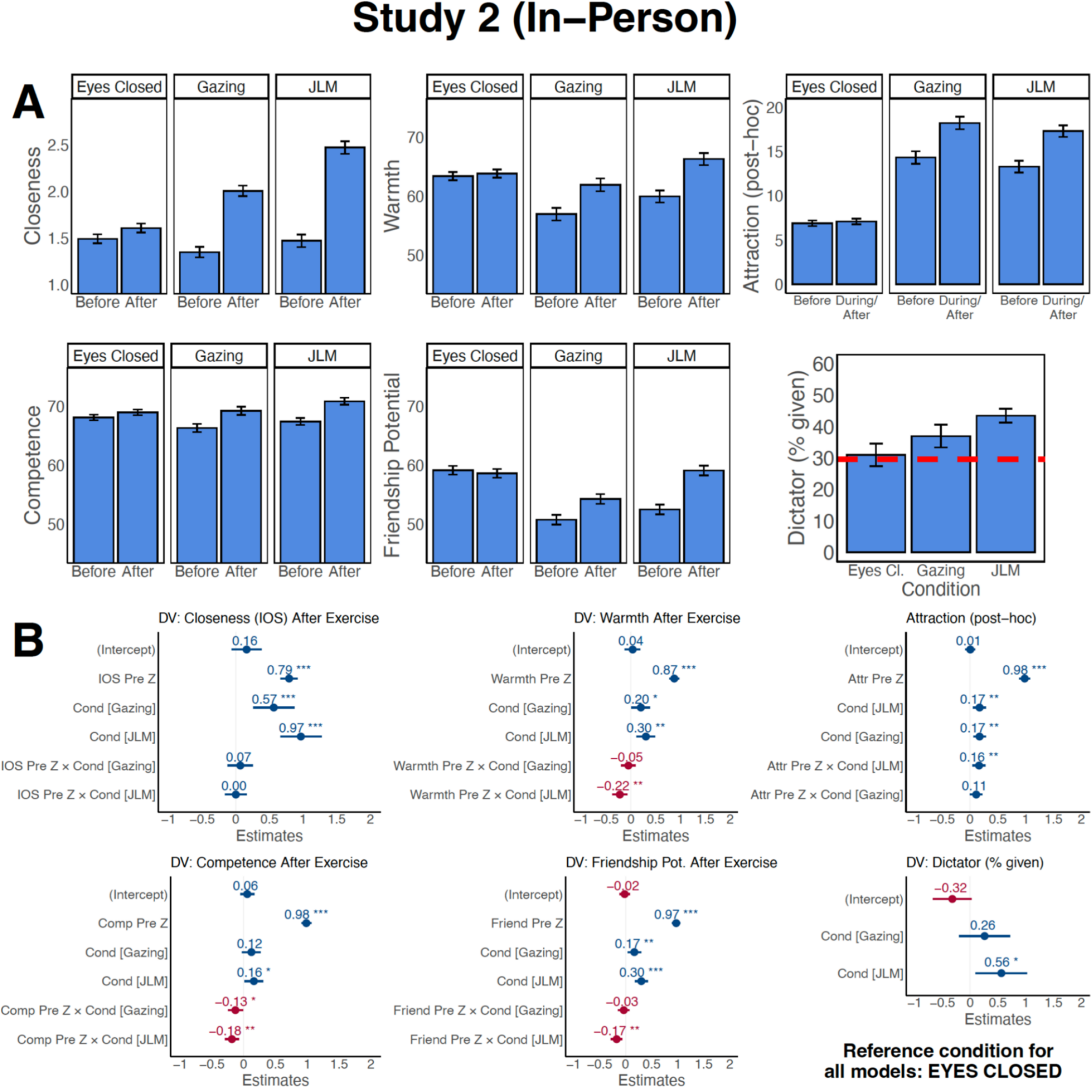
Gazing and JLM increased perceived closeness, warmth, and attraction in person, and JLM increased perceived competence and dictator game sharing. **A** Average pre-exercise and post-exercise values for the key self-report variables for both conditions, and results for the dictator game. Plots are based on individual ratings by participants per dyad. Attraction ratings were collected retrospectively rather than before and after each exercise for this study. Eyes Closed *n* = 138; Gazing *n* = 160; JLM *n* = 178. Dashed red line in dictator game plot: average proportion given in previous research (Doñate‑Buendía et al., 2022). Error bars: 95% *CI*, adjusted for within-subject designs (Loftus & Masson, 1994). Note that, for simplification and visualization, this figure treats each repeat participation as independent and treats each participant as independent from their dyad partner. For statistical testing refer to panel B. **B** Results from the mixed-effects model. Values in the figure are the standardized beta values from the model. *p < 0.05, **p < 0.01, ***p < 0.001. Eyes Closed was the reference category

JLM and Gazing increased closeness (JLM: *β* = 0.97, *SE* = 0.16, *p* < 0.001, Gazing: *β* = 0.57, *SE* = 0.15, *p* < 0.001), warmth (JLM: *β* = 0.30, *SE* = 0.09, *p* = 0.002; Gazing: *β* = 0.20, *SE* = 0.09, *p* = 0.04), friendship potential (JLM: *β* = 0.30, *SE* = 0.06, *p* < 0.001; Gazing: *β* = 0.17, *SE* = 0.06, *p* = 0.01), and attraction (JLM: *β* = 0.17, *SE* = 0.06, *p* = 0.004; Gazing: *β* = 0.17, *SE* = 0.06, *p* = 0.003) compared to Eyes Closed (Fig. 5A–B). JLM, but not Gazing, also had a positive effect on perceived competence (JLM *β* = 0.16, *SE* = 0.07, *p* = 0.03; Gazing: *β* = 0.12, *SE* = 0.07, *p* = 0.11). The effect sizes for closeness were large, with a predicted 0.97 *SD* increase for JLM and 0.57 *SD* for Gazing. The other effect sizes were small to moderate (Cohen, 1988). There were no effects of Eyes Closed, reflected by all intercepts being non-significant (*p*-values > 0.15). Pre-exercise ratings strongly predicted post-exercise ratings for all variables (e.g., closeness: *β* = 0.79, *p* < 0.001; Fig. 5B for other coefficients).

There were also interactions between JLM and pre-exercise warmth (*β* = − 0.22, *SE* = 0.07, *p* = 0.003), competence (*β* = − 0.18, *SE* = 0.06, *p* = 0.001), potential for friendship (*β* = − 0.17, *SE* = 0.06, *p* = 0.002), and attraction (*β* = 0.16, *SE* = 0.06, *p* = 0.006), as well as an interaction of Gazing with competence (*β* = − 0.13, *SE* = 0.06, *p* = 0.03). That is, for JLM, participants who initially rated their partner as incompetent, low in warmth, or low in friendship potential increased these ratings more than those who already rated their partner highly on these dimensions prior to the exercise. For Gazing, the same was true for competence. In contrast, those with *higher* pre-exercise attraction showed a relatively greater attraction increase from JLM than those with lower pre-exercise attraction.

To directly compare JLM and Gazing, we re-ran the models with Gazing (instead of Eyes Closed) as the reference condition. JLM was more effective in increasing closeness (*β* = 0.40, SE = 0.15, *p* = 0.008) and friendship potential (*β* = 0.13, *SE* = 0.06, *p* = 0.03). This was not the case for warmth (*β* = 0.10, *SE* = 0.09, *p* = 0.26), competence (*β* = 0.04, *SE* = 0.07, *p* = 0.58), or attraction (*β* = − 0.00, *SE* = 0.05, *p* = 0.996). Minor gender effects can be found in Online Resource 7, unstandardized betas in Online Resource 13.

#### Effects on Dictator Game

JLM participants shared more (*M* = 43.5%, *SD* = 15.1) than Eyes Closed participants (*M* = 31.0%, *SD* = 21.4; *β* = 0.56, *SE* = 0.24, *p* = 0.02, Fig. 5B). Gazing shares (*M* = 37.0%, *SD* = 23.4) fell between JLM and Eyes Closed, but did not significantly differ from the other conditions (Gazing vs. Eyes Closed: *β* = 0.26, *SE* = 0.23, *p* = 0.27; JLM vs. Gazing: *β* = 0.30, *SE* = 0.23, *p* = 0.19).

#### Effects on Emotions

**Fig. 6:**
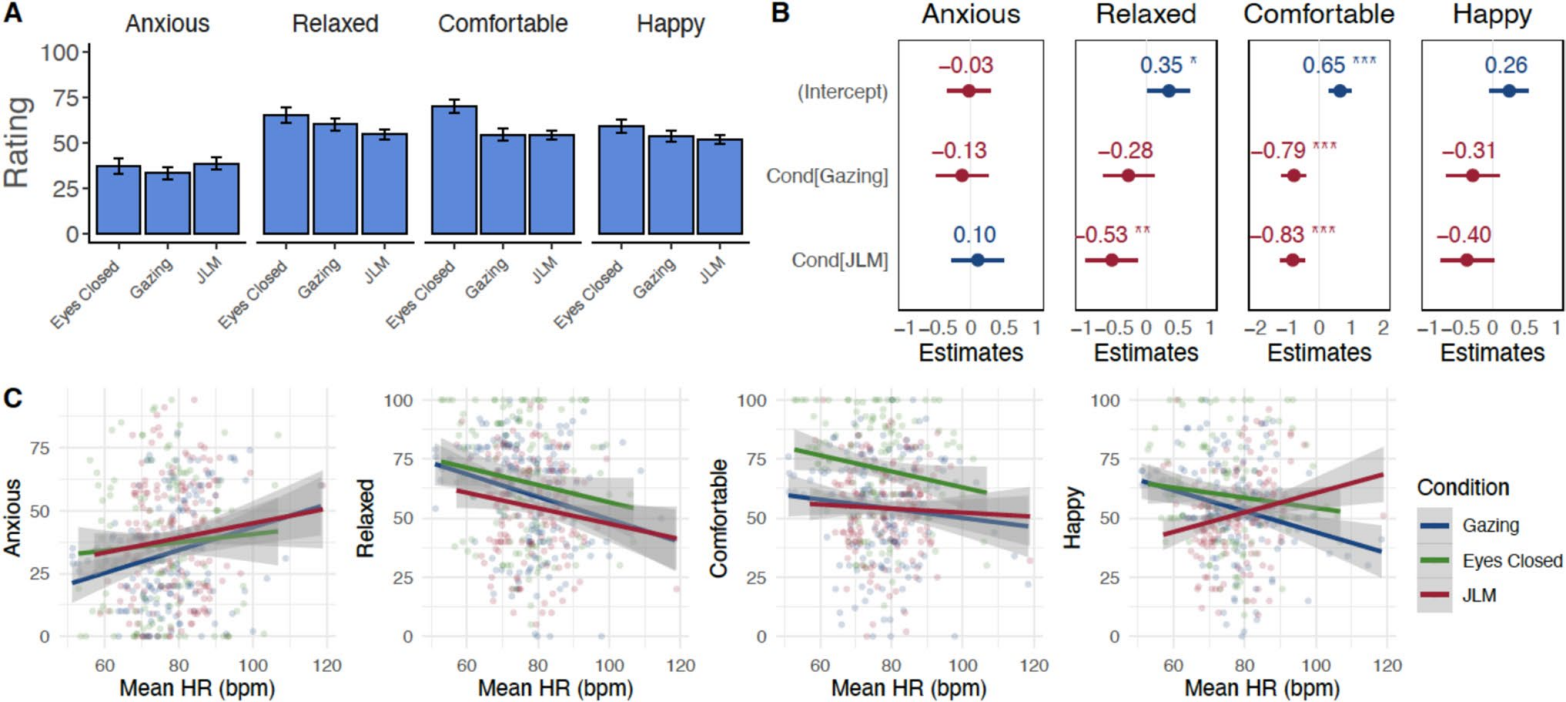
Self-reported emotions after the in-person dyads. **A** Average emotions. Plots are based on individual ratings per dyad. For visualization, this figure treats each data set as independent. For accurate statistical testing, refer to panel B. **B** Results from the mixed-effects model. Shown are the standardized beta values. Eyes Closed was set as the reference condition. ***C*** Average HR plotted against affect. Each data point corresponds to one participant in one dyad. For visualization, this plot and the linear fit lines treat each data point as independent. For accurate statistical testing, refer to the text. *Note.* Error bars denote 95% *CI*. Eyes Closed *n* = 138, Gazing *n* = 160, JLM *n* = 178. *p < 0.05, **p < 0.01, ***p < 0.001

Anxiety was low to moderate (overall *M* = 36.37 on a scale of 100, *SD* = 22.91). The positive emotions of comfort, relaxation, and happiness were rated above 50 on average (Fig. 6A). JLM participants reported feeling less relaxed (*β* = − 0.54, *SE* = 0.21, *p* = 0.011) and comfortable (*β* = − 0.83, *SE* = 0.19, *p* < 0.001) than Eyes Closed participants (Fig. 6B); Gazing participants were also less comfortable than Eyes Closed participants (*β* = − 0.79, *SE* = 0.19, *p* < 0.001). There were no significant differences between JLM and Gazing (*p*-values > 0.19), nor effects of gender composition.

#### HR Results

##### Heart Rate Predicted Self-reported Affect

Figure 6C shows average HR plotted against affect. After accounting for the nested data structure and when setting Eyes Closed as the reference, HR did not predict any of the emotions (all *p*-values for main effects of mean HR > 0.05). However, there was an interaction of mean HR with JLM (*β* = 0.39, *SE* = 0.15, *p* = 0.009): For JLM, a higher average HR predicted happiness, while for Gazing, it was linked with lower happiness. When Gazing was set as the reference, HR predicted lower relaxation (*β* = − 0.21, *p* = 0.04), and—trend-wise—higher anxiety (*β* = 0.19, *p* = 0.07). Including dyad gender composition in the models did not reveal any modulation of the effects.

##### HR Across Conditions

**Fig. 7:**
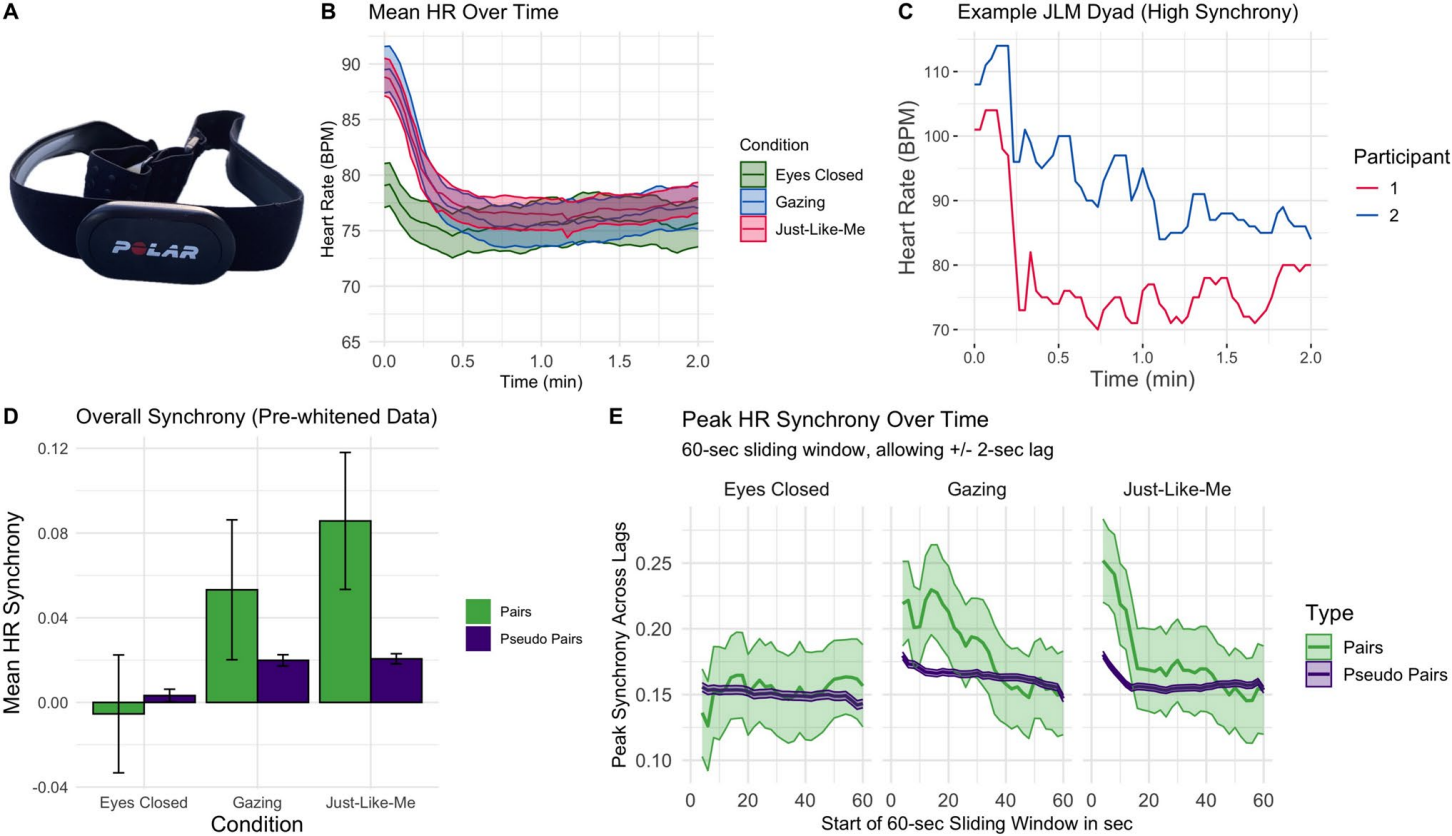
HR decreased over time, and HR synchrony was observed during the JLM meditation.**A** Polar Belt used to measure HR in mobile participants. **B** Time course of mean HR for all conditions. For simplification, this figure considers each dataset as independent. **C** Example HR time courses of two members of a JLM dyad with high synchrony. **D** HR synchrony in paired participants in dyads vs. pseudo pairs. **E** Time course of peak synchrony while allowing for a lag between responses. The *X*-axis refers to the beginning of the sliding window, up to 2 min in total. *Note*. All shaded regions and error bars: 95% CI of the mean

JLM and Gazing participants started the exercises with elevated HR (first measurement for Gazing [bpm]: *M* = 89.48, *SD* = 13.38; JLM: *M* = 88.83, *SD* = 11.45; Fig. 7A–B), which rapidly declined to stable, lower levels (after 2 min for Gazing: *M* = 77.03, *SD* = 12.06; JLM: *M* = 77.96, *SD* = 9.41). Eyes Closed participants also showed slight initial HR reductions, but from a much lower starting point (*M* = 79.06, *SD* = 11.4; after 2 min: *M* = 75.7, *SD* = 12.39). Across all conditions, the decrease in HR was highly statistically significant (JLM: *β* = − 10.92, *SE* = 0.98, *p* < 0.001; Gazing: *β* = − 12.44, *SE* = 1.34, *p* < 0.001; Eyes Closed *β* = − 3.46, SE = 0.75, *p* < 0.001). Figure 7C shows an example time course for one JLM dyad with high synchrony.

##### HR Synchrony Across Conditions

After Bonferroni correction for multiple comparisons, HR synchrony was significantly higher in JLM pairs than in JLM pseudo pairs [*t*(15,346) = 4.05, *p* < 0.001], and also higher in JLM pairs than in Eyes Closed pairs [*t*(149) = 3.9023, *p* < 0.001; required threshold = 0.008; Fig. 7D]. The other comparisons, including Gazing pairs vs. pseudo pairs [*t*(12,421) = 1.98,* p* = 0.048] and Gazing pairs vs. Eyes Closed pairs [*t*(140) = − 2.53,* p* = 0.01], did not reach significance after correction (uncorrected *p*-values reported for reference). Synchrony in JLM pairs also did not differ from Gazing pairs [*t*(167) = 1.38, *p* = 0.17], and synchrony in Eyes Closed pairs was not higher than for Eyes Closed pseudo pairs (*t*(8131) = − 0.50, *p* = 0.62). *p*-values reported here are uncorrected for reference. The time course of peak synchrony across lags (Fig. 7E) indicated that synchrony mostly occurred in the beginning of the meditations and then quickly decreased towards levels similar to pseudo pairs.

##### HR Synchrony Did Not Predict Changes in Partner Ratings and Dictator Game

While controlling for condition and mean HR, synchrony of pre-whitened HR data did not predict changes in closeness (*β* = 0.03, *SE* = 0.14, *p* = 0.81), warmth (*β* = − 0.12, *SE* = 0.16, *p* = 0.46), competence (*β* = 0.04, *SE* = 0.17, *p* = 0.80), friendship potential (*β* = 0.04, *SE* = 0.16, *p* = 0.82), or dictator game sharing (*β* = 0.10, *SE* = 0.16, *p* = 0.52), with Eyes Closed as the reference. The same was true with Gazing as the reference. Additionally, none of the interactions between HR synchrony and condition (Gazing or JLM) were significant for any outcome variables (all *p*-values > 0.15), indicating that HR synchrony did not predict any outcomes for these conditions either. Including gender composition in the models did not qualitatively change these results. Given the lack of effects, this analysis was not repeated for pseudo pairs.

#### Robustness Analysis: Exclusion of Familiar Dyads

Our results remained largely robust when analyzing only unfamiliar participants. A few small changes emerged in the competence and friendship potential analyses, as well as in the HR synchrony analysis for the Gazing condition (for details, see Online Resource 8). These changes may reflect an influence of familiarity or simply reduced statistical power. Most importantly, however, the effects of JLM on closeness and HR synchrony remained significant.

#### Textual Responses

Comments can be found in Online Resource 11. Many participants reported liking the experiment or finding it fun. Several also mentioned awkwardness again, which typically declined over time.

## General Discussion

Our study demonstrates that brief dyadic meditative exercises can produce short-term effects on interpersonal closeness, positive partner perceptions, prosocial behavior, and interpersonal synchrony in both virtual and in-person settings. Specifically, we evaluated the JLM exercise, which consists of mutual gazing between participants, accompanied by the contemplation of prompts that focus on universal feelings, needs, and desires. We compared JLM to one active dyadic control condition, Gazing, and—in Study 2 only—to a solitary meditation control condition, Eyes Closed.

In both virtual and in-person settings, JLM and Gazing substantially enhanced feelings of closeness from pre-meditation levels, showing large effect sizes in line with previous findings (Kok & Singer, 2017). Both exercises also enhanced perceptions of warmth (significant for Gazing in Study 1 and for both conditions in Study 2), attractiveness, and friendship potential (the latter assessed only in Study 2). In Study 2, JLM also fostered perceived competence, though this effect was less robust (Online Resource 8). For individuals initially perceived as particularly low in competence, warmth, or friendship potential, JLM was especially effective in improving these ratings, which is promising for practical applications. A similar effect was observed for competence in the Gazing condition. Those initially seen as more attractive experienced a greater increase in attraction ratings from JLM in Study 2, possibly because contemplating shared emotions deepened an initially more superficial attraction. The substantial effects of Gazing across both studies are noteworthy. Simply paying close attention to another person and experiencing them looking back can, under the right circumstances (Tobiasen & Allen, 1983), have significant positive impacts (Burgoon et al., 1985; Kellerman et al., 1989).

We hypothesized that JLM would have stronger effects than Gazing alone. In Study 1 (virtual context), however, no differences emerged between conditions on self-report measures. Notably, we observed greater smiling synchrony for JLM than Gazing after controlling for average smiling, suggesting subtle, non-self-reported distinctions between conditions. This finding underscores the potential value of facial affect measures in meditation and social intervention research. Controlling for average smiling was crucial, as participants in the Gazing condition smiled more on average than those in JLM, possibly due to the more serious tone of JLM prompts, which may have reduced smiling and minimized more natural laughter. In Study 2 (in-person), JLM showed significantly stronger effects than Gazing for closeness and friendship potential, though the latter was less robust (Online Resource 8). The lack of self-report differences in Study 1 might be attributed to its virtual context. Additionally, a post-hoc power analysis indicated that Study 1 was underpowered to detect small-to-moderate differences, whereas Study 2 was adequately powered—likely contributing to the contrasting results across studies.

Participants also shared more money in a hypothetical dictator game after the dyadic conditions. In Study 1, allocations to the partner exceeded those reported in prior research (Doñate‑Buendía et al., 2022). However, this comparison is limited by differences in settings and sample characteristics. In Study 2, dictator game sharing was not only higher than in previous studies but also significantly greater for JLM compared to the Eyes Closed condition, providing stronger evidence of increased prosocial behavior. This aligns with previous findings showing that dictators allocate more money when provided with personal information about recipients (Bohnet & Frey, 1999) or after engaging in conversations with them (Maalouly et al., 2024), compared to when recipients remain anonymous or before such interactions. Loving-kindness meditation has also been shown to increase sharing (Reb et al., 2010). Importantly, since we did not measure participants’ intentions or motivations, the mechanisms underlying these effects remain opaque. It is possible that increased sharing resulted from increased feelings of care, benevolence, or compassion, or heightened fairness-related motivations (see also Singer & Steinbeis, 2009). Future studies should attempt to unmask the underlying mechanisms. Altruistically motivated behavior has previously been shown to increase following socioaffective training that incorporated Affect Dyads (Böckler et al., 2018).

Studies have demonstrated that even solitary meditations can lead to more prosocial behavior (Berry et al., 2020). However, what is special about dyadic meditation is that it likely engages brain regions involved in social processing (Valk et al., 2017), and may practice specific social skills (Petzold et al., 2023). In the JLM meditation, for example, the social skills engaged are the abilities to closely attend to another person, hold eye contact with awareness, and realize that the other person has had similarly painful or positive experiences in their lives as we have had in ours. Such skills and awareness are invaluable, especially in times of widespread loneliness (Office of the Surgeon General, 2023), increased screen time (Pandya & Lodha, 2021), and polarization (e.g., Piazza, 2023). The more we perceive someone as a human being with agency, the more brain regions linked with social processing may activate, fostering prosocial behavior (Tankersley et al., 2007). In contrast, dehumanizing others—whether by cognitive reframing or averting our gaze, such as from a homeless person—may reduce empathy and the urge to help (Tausen & Fossum, 2024). JLM may offer a true antidote to such dehumanization.

In terms of mechanisms, we found significant synchrony between dyad members’ smiling (Study 1) and HR (Study 2) during the meditations. In Study 1, both Gazing and JLM participants showed smiling synchrony, but JLM exhibited greater synchrony after controlling for average smiling. In Study 2, JLM participants showed significant HR synchrony, while HR synchrony in Gazing was present only as a trend. Our findings of interpersonal synchrony point to a shared experience of emotional arousal as a mechanism underlying the connection-enhancing effects of dyadic meditations. Importantly, synchrony was significantly higher in real pairs—individuals who meditated together—than in pseudo pairs. This was true for both types of synchrony for JLM and for smiling synchrony for Gazing. Pseudo pairs are participants in the same condition who did not interact with each other and form a stringent control condition for synchrony research. This suggests that synchrony emerged from nonverbal interactions rather than exposure to the same task or environment (Palumbo et al., 2017). Of note, pseudo pairs did exhibit some degree of synchrony as well, which may reflect the shared nature of the task environment. Notably, smiling synchrony could only be assessed in 63.5% of dyads, where both members smiled at least briefly. In the remaining dyads, at least one member did not smile at all—likely reflecting their preference or tendency to remain neutral or serious during the meditations.

Dyadic synchrony has generally been considered a positive characteristic in relationships. However, optimal synchrony levels may depend on the situation, and the ability to dynamically move in and out of synchrony may be important for adaptive relating (Mayo & Gordon, 2020). For instance, in an infant-parent relationship, mid-range behavioral synchrony, in terms of HR and HR variability, is associated with more securely attached children compared to too little or too much synchrony (Beebe & Steele, 2013). Similarly, securely attached adults may synchronize their behavior less than insecurely attached adults (Feniger‑Schaal et al., 2016). Moreover, the therapist-patient relationship appears to depend on dynamic modulation in neural synchrony to foster an alliance rather than codependence (Koole & Tschacher, 2016).

To explore synchrony dynamics, we plotted the time course of peak synchrony across both conditions, allowing for response lags between participants. In Study 1, smiling synchrony appeared to largely be present throughout the exercise, with Gazing showing a slight downward trend over time. For JLM, we observed a slight decline in synchrony over time, which then recovered to an intermediate level. In Study 2, synchrony occurred primarily at the beginning of the meditation, then rapidly declined to the level of pseudo pairs. Observed declines in synchrony may indicate that participants reduced their nonverbal engagement over time, perhaps due to discomfort with sustained closeness, particularly with unfamiliar partners. It would be interesting to observe experienced dyadic meditation practitioners to assess if they show stronger and more sustained synchrony later in the exercise. Novice participants may initially establish rapport (e.g., through reciprocated smiles), which subsequently diminishes, followed by occasional re-engagement in synchrony. Notably, Wohltjen and Wheatley et al. (2021) also found that synchrony in pupil size during natural conversations *declined* over time when participants held direct eye contact, until that eye contact broke, which then increased prior to re-establishing eye contact.

The elevated HR synchrony at the start of the in-person dyadic exercises may also be related to a common HR pattern we observed. That is, participants in the JLM and Gazing conditions typically showed an initially high HR that then stabilized to a lower level. This initial increase may have resulted from nervousness or excitement associated with interacting with a new partner—as shown in our affect analysis—which then diminished. In fact, average HR tended to correlate with lower relaxation and higher anxiety, at least for Gazing. This pattern of an initially high HR throughout the session was much less pronounced in the Eyes Closed condition, which was associated with a more relaxed state. Kang and Wheatley (2017) observed peak pupillary synchrony between narrator and listener during highly emotional moments in autobiographical storytelling. This finding aligns with our finding of higher HR synchrony at the onset of the dyadic exercises, which may have triggered heightened emotional arousal.

While interpreting these synchrony results, it should be kept in mind that our HR measure was very coarse (one sample every 2 s), which only allowed for the detection of equally coarse patterns in the data—in this case dominated by the steep HR decline early on in the dyadic meditations. In particular, after HR settled to a stable level after the initial arousal subsided, our measurement approach might not have picked up enough variance in HR, which would then make it less likely for synchrony between participants to become statistically evident. Hence, it is possible that more fine-grained and prolonged measurements would result in even clearer detection of synchrony, perhaps also in later stages of the exercises. Such improved measurement would also be helpful to further delineate the dynamics of dyadic interactions.

There was support for a mechanistic effect of smiling synchrony in dyadic meditation, as we found significant links between synchronization and positive outcomes in Study 1. Dyad members who coordinated their smiling more during meditations experienced greater increases in closeness, perceived warmth, and attraction. This supports previous research indicating that nonverbal physical synchrony, including smiling synchrony, can enhance rapport (Kim et al., 2009) and perceptions such as confidence (van Swol, 2003), and that synchrony can also appear in virtual settings (Stosic, 2021). In the healthcare setting, for example, behavioral mirroring and brain-to-brain concordance between clinician and patient have been linked with reduced pain (Ellingsen et al., 2020). Another study found that higher movement synchrony in simulated clinician–patient interactions was associated with reduced patient pain and enhanced trust in the clinician (Goldstein et al., 2020). These findings support a broader view of synchrony, that moving together—whether the face or the body or the eyes—may synchronize internal physiology, thereby boosting closeness and empathy. HR synchrony, however, did not predict positive outcomes. This may reflect our coarse HR measurement technology. It is also possible, however, that HR synchrony is not directly linked with the specific self-report outcomes assessed here. That is, dyadic meditations may be associated with both HR synchrony and increases in closeness, positive partner perceptions, and prosocial tendencies, but these phenomena may not necessarily be causally linked.

Our findings indicate that reported changes in perceived closeness were not simply due to demand characteristics, such as participants’ expectations or an intention to conform. Instead, the act of synchronously smiling with another person, a non-verbal behavior associated with positive social interactions and bonding, may actively contribute to enhancing feelings of connection. Our findings offer a tangible aspect of the exercise that should be further investigated and optimized. For instance, participants could be encouraged to notice and reciprocate their partner’s facial expressions, in line with embodied mirroring exercises (Smyrnis & Ginns, 2016).

Additionally, participants who smiled more reported greater comfort during the meditation, reinforcing the validity of our facial affect measures. Notably, in the Gazing condition, but not in JLM, smiling predicted happiness. This difference may stem from the relatively serious tone of JLM, which involved minimal smiling, whereas Gazing more naturally led to spontaneous smiling and laughter, perhaps due to the absence of serious contemplation prompts. One could speculate that those who *did* smile a lot or laughed during JLM may have had more difficulty truly engaging with the exercise, potentially leading to a disconnect between smiling and happiness. For example, one JLM participant commented that “laughing becomes […] an avoidance mechanism” when one does not feel comfortable being seen by others (Online Resource 11). This hypothesis requires further testing.

Another intriguing pattern emerged for the link between HR and happiness: for Gazing, higher average HR was linked with lower happiness, whereas for JLM, higher HR correlated with greater happiness. One explanation could be that the contemplative nature of JLM, which invites empathy and emotional sensitivity, might increase arousal in a way that fosters positive feelings of connection and meaning, thus enhancing happiness. In contrast, Gazing alone may lead to heightened arousal that, without the grounding effect of reflective prompts, could be experienced as disconcerting and less positive. That is, context may shape how physiological arousal is perceived and linked to subjective affect (Schachter & Singer, 1962).

While highly effective, dyadic meditation practices should not replace solitary meditation practices but rather complement them. Social practices yield distinct effects from solitary practices, with each offering its unique benefits (Engert et al., 2017; Petzold et al., 2023; Singer & Engert, 2019). These practices also engage different underlying mechanisms (Godara et al., 2024; Valk et al., 2017). In addition, our analysis showed that dyadic interactions were accompanied by higher discomfort and lower levels of relaxation compared to the solitary Eyes Closed condition. This suggests that the benefits of brief dyadic exercises with strangers may emerge as participants navigate and internally process challenging social emotions, such as shyness or the feeling of being judged, within the context of the meditation experience.

Also, in the comments, some participants reported awkwardness and low motivation to participate, particularly in the beginning and in the virtual condition. This reticence usually diminished over time. Kok and Singer (2017) likewise reported that motivation to engage in dyads may be lower than for solitary meditation. Despite initial discomfort, however, the exercises had positive effects for most people. The majority of participants reported having enjoyed the experience at least a little bit after engaging in it (83.6% in Study 1, 88.8% in Study 2), suggesting that persevering through the initial awkwardness pays dividends.

In practice, dyadic exercises could be integrated into team-building activities or conflict resolution strategies in the workplace, helping to foster connection and mutual understanding among colleagues. In settings characterized by political polarization, these exercises may also be impactful, bridging empathy gaps and promoting constructive dialogue between individuals on different sides of an issue. Similarly, in educational settings, they could be used to enhance empathy and collaboration among students. The versatility of these exercises makes them valuable tools for enhancing communication and understanding across diverse environments.

There are some caveats that are important to consider. While the potential benefits of fostering relationships through dyadic meditations are promising, it is crucial to recognize that not everyone is comfortable with heightened intimacy at all times. Maintaining personal boundaries is essential for well-being. Moreover, in both of our studies, some participants—specifically in mixed-gender dyads—reported experiencing increased romantic or sexual feelings, which could pose challenges in professional settings or for people in monogamous relationships. These effects were small. Nevertheless, it is imperative that these exercises are conducted voluntarily, without pressure, and that participants are informed they can withdraw or modify the rules at any time. Despite these caveats, the positive outcomes of enhancing social connection likely outweigh the risks. It is important, however, to establish a conducive environment, often referred to as “creating a container,” before initiating such exercises (Downing, 2021). This includes clearly informing participants that the exercise is voluntary, setting expectations, and agreeing on specific rules before starting, thereby enhancing comfort and safety (see Online Resource 12).

The JLM exercise may offer advantages over Gazing, indicated by stronger effects on closeness and—possibly—friendship potential in Study 2, heightened synchrony for smiling and HR, and participants’ tendencies to take the exercise more seriously than Gazing—as indicated by lower overall smiling. By focusing participants on contemplating deep, empathetic thoughts, the exercise could reinforce—for instance—the shared humanity between managers, employees, or colleagues, which may have positive outcomes. Gazing at each other without further instructions may more easily lead to awkwardness and laughter in novice participants (see participant comments), although even this still appears to be beneficial. Finally, self-reported adherence was lower for JLM than the other conditions, perhaps due to its higher complexity. Future research should explore how the instructions for JLM can be further simplified. For instance, one may play the prompts via audiotape or read them, as often done in retreat settings, rather than showing them in writing.

In terms of assessing virtual vs. in-person contexts for dyadic exercises, comparing Studies 1 and 2 is challenging due to differences like the exclusion of romantic attraction ratings in Study 2, which might have lessened awkwardness; the addition of another condition in Study 2; and its larger sample size and more rounds, enhancing statistical power (see also Online Resource 13). There were, however, indications in the comments that in-person implementation was preferred by participants. In the comments, the virtual format was often described as unusual or awkward, with difficulties in connecting through screens. The inherent physical and emotional detachment in virtual interactions, exacerbated by the inability to maintain genuine eye contact and the absence of other non-verbal cues like body language, likely contributed to this experience (Nadler, 2020). Participants also reported self-consciousness, possibly due to feeling closely observed, or annoyance at their partner seemingly doing other things on the computer during the exercise (Fauville et al., 2021; Ngien & Hogan, 2023). The latter problem may be reduced if participants are highly motivated to take part in virtual meditation exercises instead of simply being assigned to it, as done in this study. Despite these issues, both in-person and virtual implementation produced positive effects. Although virtual implementation has certain downsides, it may enhance accessibility and scalability owing to reduced costs and logistical demands, such as the need for a physical space or a local instructor (Petzold et al., 2023).

### Limitations and Further Research

There are several limitations to our study. First, it focused on measuring immediate effects and mechanisms of brief meditative exercises rather than the effects of regular practice over many weeks or months, as previous studies did (Godara et al., 2021; Singer, 2016). We do not know how long the altered closeness and partner perceptions last after the dyadic meditations, or whether they would be amplified or attenuated through regular practice. Second, the study was led by authors who could have inadvertently influenced participants’ behavior, such as through non-verbal cues. Our blinding procedures were designed to avoid most experimenter effects, but the possibility of bias remains. Third, we relied on a single task, the dictator game, which primarily involves fairness and giving behavior, to assess the complex construct of prosocial behavior. Fourth, the dictator game was hypothetical rather than utilizing real money. While actual and hypothetical dictator games typically yield similar results (Ben‑Ner et al., 2008), this limitation could have affected participants’ choices. Fifth, some of our outcome measures were assessed in a format that has not been validated as such, as we opted for simple one-item scale ratings to avoid participant fatigue.

Another limitation is the absence of a fully neutral control (e.g., a waitlist control). This restricted our comparisons of measures that were only assessed after the exercises (e.g., dictator game) to relative differences between conditions rather than absolute effects. For instance, we cannot determine whether JLM and Gazing increased prosocial behavior or if Eyes Closed reduced it. However, we did collect pre-exercise values for closeness and positive partner perceptions, which generally increased for dyadic conditions and never significantly decreased for Eyes Closed, suggesting that the same may apply to the dictator game results.

As mentioned, a limitation in interpreting the HR synchrony results lies in the coarse granularity of our measurement compared to other studies (Coutinho et al., 2021). We opted for our approach because it enabled easy simultaneous measurement of multiple participants, utilizing more than ten synchronized Polar belts connected to a single tablet for data collection, thus facilitating an efficient research process. Additionally, while our smiling data was more granular, it also had limitations. Many participants exhibited few smiling events and minimal movement during the interaction, likely due to their intention to remain still. Longer timescales or investigation of more interactive dyadic practices might be useful to address this. Additionally, future studies could simultaneously measure behavioral and physiological synchrony to uncover potential relationships (Koul et al., 2023).

There are several other promising directions for future research. First, some participants found it challenging to relate to the JLM prompts (Online Resource 11). Investigating how these prompts can be tailored for specific contexts and populations could improve their effectiveness. Second, extending our findings to settings like workplaces, dating scenes, or committed relationships would broaden the exercises’ applicability. Third, the potential moderating effects of individual differences like intrinsic empathy or social anxiety on the effectiveness of these exercises deserve exploration. Additionally, cultural nuances, such as the distinct interpretations of eye contact among Japanese versus US or European individuals (Argyle et al., 1986; Uono & Hietanen, 2015), warrant careful consideration. Fourth, conducting studies to explore the long-term impacts of these exercises are important. This includes assessing whether consistent practice boosts their effectiveness or results in habituation, and examining the effectiveness of JLM in mitigating the dehumanization of others. Here, it would be beneficial to include additional ecologically valid measures of prosocial behavior beyond the dictator game, and to measure underlying motivations, such as altruistic intent or fairness considerations. Fifth, based on the “shared network hypothesis” of empathy (McCall & Singer, 2013), one may predict that dyadic meditations, like JLM, activate similar brain regions in both meditation partners as they engage in shared emotional experiences. Brain imaging studies could address this hypothesis.

Finally, exploring the role of synchrony in various dyadic practices could reveal whether consistent synchrony is universally necessary for beneficial outcomes or if some practices thrive on more variable or even asynchronous dynamics (Mayo & Gordon, 2020). For instance, in practices like contemplative dyad meditation, where one partner mindfully listens to the other express their feelings or sensations, perhaps lower synchrony would be desirable—fostering a space where one can support and “hold space” for another without fully merging with the partner’s emotional state. In fact, practitioners are often explicitly instructed to maintain a neutral, compassionate facial expression instead of reacting (Anliker, 2020). Despite this directive, it is, however, also conceivable that a form of synchrony may still emerge through the deeper connection established during the practice. These future directions would deepen our understanding of how dyadic exercises can foster interpersonal connection and well-being.

To summarize, our study provides evidence that simple dyadic exercises can enhance feelings of interpersonal connection and prosocial behavior both virtually and in person, and that these impacts are mediated by interpersonal synchronization between dyad partners. Our findings highlight the value of brief dyadic meditation exercises to foster social connections in times of widespread loneliness (Lin, 2023; Na et al., 2023).

## Supplementary Information

Below is the link to the electronic supplementary material.

Supplementary file1 (PDF 559 KB)

## Data Availability

Due to the risk of participant re-identification, which could not be mitigated by simply removing specific variables, the data cannot be publicly shared. However, researchers interested in accessing the data are welcome to contact us to discuss secure access options. Any further materials or analysis scripts are either included in the Supplementary Materials or available upon request.

## Declarations

### Use of Artificial Intelligence

ChatGPT-4 (OpenAI, 2023) and ChatGPT-4o (OpenAI, 2024) served as assistive tools for debugging R analysis scripts, drafting specific analysis script or text sections, and improving language flow. The artificial intelligence (AI) output underwent rigorous review, extensive editing, and validation by VUL. For certain sections, after a thorough back-and-forth exchange with the AI, the final output was deemed suitable for inclusion almost as is. The AI also provided suggestions for visualization, analysis, literature, and interpretation. These were thoroughly evaluated and selectively integrated into the final manuscript. We acknowledge the inherent limitations and risks associated with AI tools; thus, we have employed a careful and critical approach to leverage this technology responsibly. The use of ChatGPT was intended to complement and extend human intelligence, with the goal of enhancing the overall quality and rigor of our research.
