# Supplementary material for: Synchronous Smiles and Hearts: Dyadic Meditations Enhance Closeness and Prosocial Behavior in Virtual and In-Person Settings
Source: Mindfulness (N Y). 2025 May 19;16(6):1719–44. doi: 10.1007/s12671-025-02588-7 (PMC12170796; doi:10.1007/s12671-025-02588-7)
Supplement: Supplementary file 1 — Supplementary file1 (PDF 559 KB) [file 12671_2025_2588_MOESM1_ESM.pdf]

**Supplementary Materials**

# Synchronous Smiles and Hearts: Dyadic Meditations Enhance Closeness and Prosocial Behavior in Virtual and In-Person Settings

Vera. U. Ludwig[1,2,3*], Lana Prieur[1,2], Scott M. Rennie[1,2,4], Andrew Beswerchij[1,2], Devora Weintraub[1,2], Blaire Berry[2,5], Jenny Wey[2,6], Katelyn Candido[1,2], Michael L. Platt[1,2,7]

[1]Department of Neuroscience, Perelman School of Medicine, University of Pennsylvania, US

[2]Wharton Neuroscience Initiative, the Wharton School of Business, University of Pennsylvania, US

[3]Positive Psychology Center, University of Pennsylvania, Philadelphia, US

[4]Champalimaud Centre for the Unknown, Champalimaud, Foundation, Lisbon, Portugal

[5]Department of Marketing, McCombs School of Business, University of Texas, Austin, US

[6]Department of Psychological Science, Pomona College, Claremont, US

[7]Department of Psychology, School of Arts and Sciences, University of Pennsylvania, US

*Corresponding author

**Table of Content**

Table of Content ........................................................................................................ 1

Online Resource 1: Additional Procedures........................................................................ 2

Online Resource 2: Further Information on Measures ..................................................... 5

Online Resource 3: Attention Checks................................................................................ 8

Online Resource 4: Post-Hoc Power Analysis.................................................................. 9

Online Resource 5: Modeling and Analyses Details ....................................................... 12

Online Resource 6: Further Details for Facial Affect Coding ........................................ 14

Online Resource 7: Supplemental Analysis of Dyad Gender Composition Effects ..................... 14

Online Resource 8: Robustness Analysis Excluding Familiar Participants.................................. 16

Online Resource 9: Group Composition for Study 1 ........................................................ 23

Online Resource 10: Group Composition for Study 2 ...................................................... 24

Online Resource 11: Comments by Participants .............................................................. 25

Online Resource 12: Tips for the Practical Implementation of the Exercises .............................. 32

Online Resource 13: Unstandardized Betas....................................................................... 33

Supplemental References.................................................................................................. 35

## Online Resource 1: Additional Procedures

### Additional Procedures for Study 1

### Script Used in the Main Zoom Room

After all logistical and technical aspects were taken care of and no more participants were expected to come into the Zoom room, the main experimenter started to officially welcome the entire group and gave overall instructions. She followed the following script:

> *"Welcome everyone! In today's study, you will do brief meditative exercises with different partners. Each meditation exercise takes 2 minutes, and this will be repeated four times. We are interested in what effects these meditations have on you. You will be assigned to a breakout room with a partner. In each breakout room, an assistant will be present, but they won't be on camera - they'll give instructions via microphone.*
>
> *In the breakout rooms, there are three phases: first you complete a questionnaire about your first impression of the other person, who is in the room with you. When you are both ready, let the experimenter know via chat or speaking. Second, the meditation exercise begins. The instructions for the exercise will be shown on your screen. Make sure to read it carefully and remember what you are supposed to do. Then switch to the view in which you see the other person (in full screen) and follow the instructions for 2 min. The experimenter will start a timer of 2 min when you are both ready and record you during those minutes. The experimenter will notify you when 2 min have passed. Then there is 'after questionnaire'. It is ok to laugh, move, or react to each other, if that happens naturally, all is fine. You don't have to sit there stiff. But please don't talk to each other and keep your microphones muted."*

After asking if there were any questions, participants were assigned to breakout rooms in pairs. If there was an odd number of participants, one participant per round stayed with the main experimenter and waited for the next round.

### Procedure Within Each Breakout Room

In each round, assistants in the breakout rooms waited for two participants to be assigned to their room. They then greeted participants via microphone, made sure that participants had speaker view activated and that they started to answer the questions that were due *before* each exercise (pre-exercise ratings of partner). Assistants waited for both participants to finish these and to silently read the instructions for the exercise. Then they announced the start of the exercise, started a 2 min timer and started to record the interaction on video. After 2 min passed, assistants reminded participants to answer the questionnaires due *after* each exercise (another rating of partner, reporting emotions, dictator game). When all dyads were done with the round, participants were assigned a new partner.

### Exercise Instructions

The instructions for the conditions were shown in Qualtrics on the screen before participants started the exercise in each round, as follows:

1. **Just-Like-Me (JLM)**

   *You will now look at each other for 2 minutes. You will be recorded. The moderator will let you know when to start and when to end.*

   *Please simply look at the other person's face while contemplating the following sentence:*

   ***[sentence prompt, see main manuscript]***

2. **Gazing**

    *You will simply look at each other (face/eyes) for 2 minutes. You will be recorded. The moderator will let you know when to start and when to end.*

    ***Please simply look at the other person's face or eyes for 2 minutes.***

## Additional Procedures for Study 2

### Exercise Instructions

In Study 2, the instructions for the conditions were again shown in Qualtrics on the screen before participants started the exercise in each round, as follows:

1. **JLM**

    *You and your partner in this round will look into each other's eyes for 2 minutes without speaking,* ***while contemplating a sentence****.*

    *Don't worry if you have to smile or laugh, or even if you have to look away for a bit. Just try your best to keep eye contact and focus on the sentence (for example, you can repeat it in your head).*

    *The moderator will let you know when to start and when to end.*

    *Please look into the other person's eyes while contemplating the following sentence:*

    *[sentence prompt, see main manuscript]*

2. **Gazing**

    *You and your partner in this round will look into each other's eyes for 2 minutes without speaking. Don't worry if you have to smile or laugh, or even if you have to look away for*

*a bit. Just try your best to keep eye contact. The moderator will let you know when to start and when to end.*

*Please look into the other person's eyes for 2 minutes.*

**3. Eyes Closed**

*In this exercise, we simply ask you to close your eyes and observe your own breath for 2 minutes. The moderator will let you know when to start and when to end. Don't worry if you have to smile or laugh.*

*Please just sit with your eyes closed for 2 minutes.*

## Online Resource 2: Further Information on Measures

### Dictator Game Instructions for Both Studies

The instructions used for the dictator game were as follows: *"*Imagine we would give you $100. You can decide how to distribute it amongst you and your partner. Please choose how much you would give to the other person and how much you want to keep for yourself. Please don't talk about this with your partner. You will be asked this question after each interaction, and your partners will not know who gave them something and who did not." The response was given on a slider from 0 ["give $100 to partner (keep 0)"] to 100 ["keep $100 (give 0)"].

### Motivation Measure for Both Studies

In Study 1, the motivation question for both conditions was "How motivated are you to try today's experiment, which involves doing small meditation exercises in breakout rooms with people you might not know? Your payment or participation does not depend on your answer -

we'd just like to know how you feel.” Options were “Not at all motivated / don't really feel like doing it, but will give it a try”, “Somewhat motivated”, ”Very motivated / really feel like doing it.” Participants were reminded that their payment or participation did not depend on the answer. In Study 2, the question for all three conditions was: “How motivated are you to try today's experiment, which involves doing small meditation exercises with people you might not know? Your payment or participation does not depend on your answer - we'd just like to know how you feel.” Options were equivalent to those in Study 1.

## Meditation Experience for Both Studies

We asked participants how often they engaged in any sort of meditation [“Very often (daily)”, “Often (a few times each week)”, ”Sometimes (a few times each month)”, ”Rarely (less than once a month)”, “Never or almost never”]. We also asked whether participants had “ever taken part in meditation exercises that involved meditating together with another person? (dyadic meditations)” [“yes, namely”, “no”, or “I don’t know”].

## Mask Wearing in Study 2

At the time of the study, mask wearing regulations from the pandemic had only just been rescinded and mask use was voluntary. We therefore also asked “Did you wear a face mask during the exercises today?” with four response options: “No, I never wore a face mask during the exercises”, “I briefly wore a face mask during the exercises, but most of the time I did not” , ”I wore a face mask during most of the exercises, but not the entire time”, and ”I wore a face mask during all of the exercises”.

## Adherence Measure in Study 2

We included the question “When looking back to all the rounds in which you had a partner, to what extent were you able to follow the instructions in the exercises?” with the response options “I followed the instructions” with a slider from 0 (not at all) to 100 (completely), starting with a default of 50. There was also the option to provide an open comment.

## Attraction Measure in Study 2

In Study 2, we queried attractiveness after all experimental rounds were finished, as follows:

> *“For our research, it is useful to know if you experienced any romantic and/or sexual attraction to your partner in each round, and whether you think the exercise increased that attraction.*
>
> *Don't worry, romantic-sexual attraction is completely natural so we will not judge. We will keep this confidential and we won't let your partners know.*
>
> *Note that indicating romantic and/or sexual attraction here does not mean that you would be interested in pursuing this interest. For example, you may be in a committed partnership and not interested in anyone besides your real partner--and nevertheless you might experience some romantic and/or sexual attraction to someone else.*
>
> *Also note that saying that you were "not attracted at all" to someone does not imply that you thought the other person was ugly or not attractive in general - just that you did not experience attraction to this person today.*
>
> *Please be as honest as you can.*
>
> *If you don't quite remember who was your partner in each round, you may guess who was who, and indicate in the next question that you do not quite remember the order.”*

*[slider from 0 to 100: 0: I was not attracted at all, 10: Extremely attracted; or "Did not have partner/round or prefer not to answer".*

Each round was listed separately, and there were two items for each round (before and during/after the exercise). At the end, we also asked "When answering the previous question, do you remember the order of your partners sufficiently?" Response options were "Yes, I remember the order exactly," or "I am not sure I remember the order exactly right."

### Other Measures

Participants also completed the *Interpersonal Reactivity Index* (IRI), and they reported emotions they perceived in their partners. This included the same emotion ratings as they completed for themselves. These variables were collected for a different research project with a focus on empathy. They are therefore out of scope for this paper and are not reported.

## Online Resource 3: Attention Checks

### Attention Checks Included in the Survey

In the Qualtrics, a focus question was included to ask if participants paid attention. Options were "I paid attention to all questions and answered honestly" (ideal answer), "I paid attention sometimes, but usually not" (considered an acceptable answer if the other attention check was passed), "I was not paying any attention" (unacceptable), "I skipped through most of the questions without reading" (unacceptable), and "I tried to "beat" the survey by answering dishonestly" (unacceptable). We also hid one further attention check in the IRI questionnaire ("Please select '3' to show that you are paying attention.").

### Results for the Attention Checks in Study 1

Noone got both focus/attention checks wrong so no participants were excluded based on this. However, two participants self-reported that they "paid attention sometimes, but usually not." All participants were considered to have passed the other attention check ("Please select '3' [...]."). However, only 80% picked the option labeled with "3" upon reading the request. The other 20% all picked the third option in the list, labeled as "2." This may be due to inattention, but more likely due to misunderstanding the item, since many participants made the same error.

### Results for the Attention Checks in Study 2

Again, no participant got both focus/attention checks wrong so no one was excluded based on this. However, yet again, two participants reported that they "paid attention sometimes, but usually not". For the attention check, ("Please select '3' [...]."), 60% picked "3," and 40% picked "2" (the third option in the list), again indicating that our instructions for this attention check were not clear. Everyone was counted as "passed."

## Online Resource 4: Post-Hoc Power Analysis

### Method for Power Analysis

We conducted a simplified post-hoc power analysis to determine the minimum detectable effect sizes for both within-condition and between-condition comparisons, adjusting for repeated measures and participant clustering. We based this estimation on one of our key outcome variables, closeness increases during the exercise (i.e., Closeness after the exercise minus before the exercise). Closeness was selected because it directly reflects the central goal of the interventions: enhancing interpersonal connection. Although the study included other variables,

such as perceptions of warmth, competence, attractiveness, and dictator game sharing, we focused on closeness as the key outcome variable to estimate statistical power, given its relevance to the primary hypotheses.

First, we calculated the intraclass correlation coefficient (*ICC*) for each condition (JLM and Gazing) by fitting linear mixed models that included both participant and partner as random effects (1). Partner was omitted for the Eyes Closed condition in Study 2, since the variance explained by the partner was 0 and including it led to singular fit issues. Using the *ICC*-values, we adjusted the sample sizes to account for the average number of repetitions per participant. We then conducted power analyses using the *pwr* package in R (2). For Study 1, we assumed one-sample t-tests for detecting effects within each condition and independent t-test for detecting differences between the conditions. For Study 2, we assumed one-sample t-tests again for within-subject effects. Given the presence of three groups, we assumed a one-way ANOVA for group differences. The harmonic mean of the adjusted sample sizes across all conditions was entered as the sample size per group in this case. T-tests and one-way ANOVA were used for simplicity in the power analyses, as they provide a reasonable approximation of power for detecting differences in the outcome variable. More complex power analyses may require the use of simulations, which rely on certain assumptions. The current procedures were deemed a useful, practical approximation of achieved power for our mixed design. We set the desired power to 0.80 and the significance level at $\alpha = 0.05$.

## Results for Power Analysis

For Study 1, original sample sizes (in terms of individual data sets from all dyads) were 102 for JLM and 98 for Gazing, including repeated measures per participant (3.78 repetitions on average for JLM and 3.5 for Gazing). The *ICC* for participants' closeness difference scores was 0.28 for JLM and 0.46 for Gazing, indicating moderate consistency across repeated measures within individuals. The *ICC* for partners was small with 0.13 for JLM and 0.06 for Gazing, indicating that specific partners did not have strong consistent effects on other participants. Adjusted for repeated-measures, the effective sample sizes were 57.31 for JLM and 45.65 for Gazing.

For Study 2, the original sample sizes were 178 for JLM, 160 for Gazing, 138 for Eyes Closed, including repeated measures per participant (5.39 repetitions on average for JLM, 4.57 for Gazing, and 4.60 for Eyes Closed). The ICC for participants was 0.42 for Just Like Me, 0.32 for Gazing, and 0.35 for Eyes Closed, again reflecting moderate consistency across repeated measures. The *ICC* for partners was small, at 0.04 for Just Like Me and 0.06 for Gazing, with no detectable partner effect in the Eyes Closed condition. The resulting effective sample sizes were 62.11 for Just Like Me, 75.03 for Gazing, and 61.19 for Eyes Closed.

For Study 1, the minimum detectable effect size for within-subject effects was $d = 0.38$ (Cohen's d) for JLM, and $d = 0.42$ for Gazing [reference values are small: $d = 0.2$, medium: $d = 0.5$, large: $d = 0.8$ (3)]. Hence, the study is powered to detect moderate effects of the exercises on closeness. This is sufficient, as previous research has shown large effects of dyadic meditations on closeness (4). For the between-group comparison (JLM vs. Gazing), the minimum detectable

effect size was $d = 0.56$. Hence, the study is powered to detect medium-to-large group differences. Smaller group differences may not be captured with the Study 1 sample size.

For Study 2, the minimum detectable effect sizes for within-subject effects were $d = 0.36$ (Cohen's $d$) for JLM, $d = 0.33$ for Gazing, and $d = 0.36$ for Eyes Closed. For comparisons between groups, the minimum detectable effect size was $f = 0.22$ [Cohen's $f$, reference values are small: $f = 0.10$, medium: $f = 0.25$, large: $f = 0.40$ (3)]. Hence, Study 2 is well-powered to detect small-to-moderate effects for both within-condition effects and group differences between conditions for the closeness outcome variable.

## Online Resource 5: Modeling and Analyses Details

### General Information on Modeling

We used the R package *lmerTest* (5) with the Satterthwaite method to calculate *p*-values and lmerControl optimizer set to Nealder-Mead. In each model, we initially included random intercepts for participant ID, dyad ID, partner ID, and experimental round [(1|DyadID) + (1|SubjectID) + (1|PartnerID) + (1|Round)]. However, including all random intercepts typically led to singular fit issues. To address this, we removed the worst-performing random intercepts (in terms of variance) per model (see sections below). Removing them did not qualitatively change the results on any occasion. We also did not include random slopes since this led to further issues fitting the models and since there was no strong theoretical reason to do so.

### Specifics for Different Models

***Study 1 - Closeness, Competence, Warmth, and Attractiveness.*** The random intercept for Round was excluded for the models on closeness, competence, and attractiveness, and SubjectID was excluded for warmth.

***Study 1 - Dictator Game.*** Random intercepts of dyadID and partnerID were not included.

***Study 1 - Self-Reported Emotions***. We initially included random intercepts for participant ID, dyad ID, partner ID, and experimental round. However, dyad ID was removed from the models for anxiety, comfort, and relaxation due to singular fit issues and variance close to 0; and partner ID was removed for relaxation. For the models exploring the link between smiling and affect, dyad ID was excluded for anxiety and comfort. For happiness, partner ID was excluded, and for relaxation, partner ID and dyad ID were excluded.

***Study 2 - Closeness, Warmth, Competence, Friendship, and Retrospective Attraction.*** The random intercept for Round was omitted for the models on closeness and competence. For attraction, only retrospectively reported values were available, meaning participants might misremember the partner order. We thus only added a random intercept for participant ID.

***Study 2 - Dictator Game.*** The random intercept for PartnerID and DyadId were omitted.

***Study 2 - Self-Reported Emotions***. For the model on comfort, the random intercept for partnerID was excluded and for the model on happiness, experimental round was excluded, neither of which changed the results. For the models exploring the link between average heart rate and affect, partnerID was excluded for anxiety and comfort, and dyad ID was excluded for happiness.

***Study 2 – Raw HR Change.*** Random intercepts for experimental round were removed across all conditions, and partner ID was removed in the Gazing condition. Variance estimates for these were effectively zero, and including them led to singular fit issues.

## Online Resource 6: Further Details for Facial Affect Coding

The recorded videos showing two participants side-by-side were cropped into halves via Bonsai (6) and/or I-movie, resulting in one person depicted per video. Data were then processed using Affectiva AFFDEX version 5.1. Affectiva provides a range of outcome variables (e.g., smiling, and movements of specific muscles). The recordings contained minimal facial movement, as most participants maintained neutral expressions. Some, however, began smiling at each other or even laughing. We rounded values to one decimal place to maintain meaningful granularity while minimizing measurement noise. The data, sampled at 25 Hz, were also temporally smoothed over a 160 ms window by applying a rolling mean using the *zoo* package in R, to highlight genuine brief changes in facial expression and filter out noise (7,8).

## Online Resource 7: Supplemental Analysis of Dyad Gender Composition Effects

### Analytical Approach

It is possible that results differ depending on the gender composition of the dyad. Therefore, in exploratory analyses we assessed whether models qualitatively changed when a mixed-gender (vs. same-gender) dummy variable was introduced into the models, including interaction effects with the main predictors.

### Results for Study 1

***Closeness, Warmth, Competence, and Attractiveness.*** Whether dyads were mixed-gender or same-gender had no significant effect on outcomes.

***Dictator Game.*** There was no statistically significant effect of gender mix of dyads.

***Smiling Synchrony and Partner Ratings.*** For attractiveness only, there was a significant interaction between mixed-gender dyads and smiling synchrony ($\beta = 0.62$, $SE = 0.27$, $p = 0.03$), indicating that in mixed dyads—but not in same-gender dyads—synchronized smiling was associated with stronger increases in attractiveness. There were no other effects of dyad gender composition. Note that we controlled for condition and average smiling in this model; however, we removed interactions between average smiling and gender composition or condition as well as any higher-level interactions. Including these led to excessively high VIF-values indicating problematic multicollinearity, which can distort coefficient estimates and make it difficult to isolate the effects of individual predictors.

***Smiling Behavior and Dictator Game Responses.*** Gender composition had no effects in this analysis. Note, however, that we again had to remove some of the interaction effects, meaning that this model was not entirely equivalent to the model in the manuscript.

## Results for Study 2

***Closeness, Warmth, Competence, and Friendship Potential.*** No significant main or interaction effects involving dyad gender composition were found except for the following: For closeness, there was a three-way interaction of Pre-Exercise Closeness with JLM and mixed-gender ($\beta = 0.40$, $SE = 0.19$, $p = 0.036$). Mixed-gender dyads showed a steeper relationship of pre-exercise closeness with post-exercise closeness than same-gender dyads for JLM, while same-sex dyads showed a steeper relationship for Gazing and Eyes Closed. For competence, there was an interaction of Gazing with mixed-gender ($\beta = 0.24$, $SE = 0.11$, $p = 0.04$), and a three-way interaction of pre-exercise competence with Gazing and mixed-gender ($\beta = -.21$, $SE = .11$, $p = 0.04$). That is, for Gazing, mixed-gender dyads reported higher competence ratings post-exercise

compared to same-gender dyads, while there was no difference for Eyes Closed and JLM. Moreover, for Gazing, mixed-gender dyads with low pre-exercise competence ratings gave relatively higher competence ratings after the exercise compared to same-gender dyads or dyads with high pre-exercise competence ratings.

***Retrospective Attraction.*** For attraction, only retrospectively reported values were available, meaning participants might misremember the partner order (i.e., which partner they meditated with in each round they reported on). For this reason, we did not explore gender composition effects for attraction.

***Dictator Game.*** There were also no effects of dyad gender mix.

***HR Synchrony Across Conditions.*** Whether dyads were same-gender or mixed-gender did not affect HR synchrony.

## Online Resource 8: Robustness Analysis Excluding Familiar Participants

### General Approach

Since familiarity between meditation partners may be related to key study outcomes such as closeness and synchrony, it could act as a confounding factor. We therefore assessed the robustness of our main results by excluding datasets where a participant reported familiarity. In Study 1, this applied to seven out of 200 individual datasets (3.5%); with five dyads out of 101 dyads including at least one participant reporting familiarity. In Study 2, 31 out of 476 individual datasets (6.5%) were excluded, impacting 17 out of 238 dyads. In the synchrony analyses, exclusion was applied only to real pairs, as familiarity levels were unavailable for all pseudo pairs. Additionally, since pseudo pairs were not actual dyads, excluding them based on familiarity would lack meaningful interpretation.

We repeated all key analyses in the reduced samples. We chose this approach rather than including familiarity as a model predictor, as the small number of familiar cases could lead to unreliable parameter estimates due to increased variability and disproportionate influence from individual cases. The number of familiar cases was also too low for a reliable direct comparison between familiar and unfamiliar individuals. Below, we present tables comparing key effects from the original analyses with those from the reduced dataset. The "Change in Significance" column indicates whether any effects in the updated analyses crossed the $\alpha = 0.05$ threshold, either becoming significant or no longer meeting the significance criterion.

## Results of Robustness Analysis

### Study 1: Closeness, Warmth, Competence, Attractiveness, and Dictator Game After Excluding Familiar Individuals

The findings for partner ratings and dictator game decisions in Study 1 remained virtually identical after excluding familiar individuals (Table S1). The only change was that the interaction of pre-exercise closeness with JLM was no longer significant. This could possibly suggest that the interaction was driven by familiarity rather than pre-exercise closeness per se. However, we cannot draw any firm conclusions given that there were only five dyads reporting familiarity. Overall, excluding familiar individuals did not meaningfully influence the estimated effect sizes and significance levels.

### Study 1: Smiling Synchrony After Excluding Familiar Individuals

Almost all results for smiling synchrony remained highly consistent after excluding dyads reporting familiarity. In the general synchrony analysis (Table S2), minor shifts included slightly smaller effect sizes and marginally higher p-values for two interactions, both of which remained

significant. All other effects were virtually unchanged. Likewise, all effects regarding the predictive power of smiling synchrony for partner perceptions and dictator game decisions remained robust (Table S3). In sum, the smiling synchrony results were not confounded by familiarity. However, given the small number of dyads reporting familiarity, these results should not be interpreted as evidence that familiarity has no influence on synchrony-related effects.

**Table S1: Partner Rating Analysis After Excluding Familiar Individuals in Study 1**

| Outcome Variable | Predictor | Original Estimate (*SE*, *p*) | Updated Estimate (*SE*, *p*) | Change in Significance |
|---|---|---|---|---|
| Closeness | Gazing (Intercept) | 1.23 (0.26, < 0.001) | 1.24 (0.26, < 0.001) | No |
| | JLM (> Gazing) | 0.06 (0.36, 0.86) | 0.04 (0.37, 0.92) | No |
| | Pre-Exercise Value | 0.49 (0.20, 0.02) | 0.53 (0.21, 0.01) | No |
| | Pre-Exercise Value × JLM | 0.51 (0.25, 0.04) | 0.36 (0.28, 0.19) | Yes |
| Warmth | Gazing (Intercept) | 0.28 (0.09, 0.008) | 0.29 (0.09, 0.008) | No |
| | JLM (> Gazing) | -0.18 (0.12, 0.15) | -0.19 (0.12, 0.13) | No |
| | Pre-Exercise Value | 0.88 (0.09, < 0.001) | 0.89 (0.09, < 0.001) | No |
| | Pre-Exercise Value × JLM | -0.06 (0.11, 0.61) | -0.08 (0.11, 0.48) | No |
| Competence | Gazing (Intercept) | 0.08 (0.06, 0.19) | 0.08 (0.06, 0.20) | No |
| | JLM (> Gazing) | -0.06 (0.09, 0.49) | -0.06 (0.09, 0.50) | No |
| | Pre-Exercise Value | 0.96 (0.06, < 0.001) | 0.96 (0.06, < 0.001) | No |
| | Pre-Exercise Value × JLM | -0.03 (0.08, 0.67) | -0.04 (0.08, 0.63) | No |
| Attractiveness | Gazing (Intercept) | 0.17 (0.08, 0.04) | 0.17 (0.08, 0.04) | No |
| | JLM (> Gazing) | 0.06 (0.11, 0.60) | 0.05 (0.12, 0.64) | No |
| | Pre-Exercise Value | 0.79 (0.06, < 0.001) | 0.78 (0.07, < 0.001) | No |
| | Pre-Exercise Value × JLM | 0.01 (0.08, 0.95) | -0.01 (0.09, 0.94) | No |
| Dictator Game | (Intercept) | 0.11 (0.17, 0.53) | 0.11 (0.17, 0.53) | No |
| | JLM (> Gazing) | -0.18 (0.24, 0.47) | -0.16 (0.25, 0.51) | No |

*Note*. "Pre-exercise value" refers to the reported value on the respective outcome variable prior to starting the meditative exercise. *SE*: standard error.

**Table S2. Smiling Synchrony Analysis After Excluding Familiar Dyads in Study 1**

| Effect | Original Estimate (*SE*, *p*) | Updated Estimate (*SE*, *p*) | Change in Significance |
|---|---|---|---|
| (Intercept) | -0.02 (0.02, 0.26) | -0.02 (0.02, 0.26) | No |
| JLM (> Gazing) | 0.13 (0.03, < 0.001) | 0.13 (0.03, < 0.001) | No |
| Pairs vs. Pseudo Pairs | 0.86 (0.19, < 0.001) | 0.84 (0.19, < 0.001) | No |
| Smiling Mean | -0.02 (0.02, 0.15) | -0.02 (0.02, 0.15) | No |
| JLM × Pairs | 0.70 (0.31, 0.02) | 0.62 (0.31, 0.049) | No |
| JLM × Smiling Mean | 0.23 (0.05, < 0.001) | 0.23 (0.05, < 0.001) | No |
| Pairs × Smiling Mean | 0.31 (0.12, 0.009) | 0.32 (0.12, 0.008) | No |
| JLM × Pairs × Smiling Mean | 0.88 (0.39, 0.02) | 0.79 (0.39, 0.04) | No |

*Note*. *SE*: standard error.

**Table S3. Smiling Synchrony Predicting Partner Ratings and Dictator Game Decisions After Excluding Familiar Dyads in Study 1**

**Partner Perceptions**

| Predictor | Original Estimate (*SE*, *p*) | Updated Estimate (*SE*, *p*) | Change in Significance |
|---|---|---|---|
| Smiling Synchrony → Closeness | 0.56 (0.19, 0.004) | 0.57 (0.19, 0.003) | No |
| Smiling Synchrony → Warmth | 0.55 (0.19, 0.006) | 0.54 (0.19, 0.006) | No |
| Smiling Synchrony → Competence | 0.07 (0.17, 0.70) | 0.08 (0.17, 0.66) | No |
| Smiling Synchrony → Attractiveness | 0.25 (0.20, 0.23) | 0.24 (0.20, 0.24) | No |

**Dictator Game**

| Predictor | Original Estimate (*SE*, *p*) | Updated Estimate (*SE*, *p*) | Change in Significance |
|---|---|---|---|
| Smiling Synchrony → Dictator Game | 0.18 (0.19, 0.36) | -0.17 (0.19, 0.39) | No |
| Smiling Synchrony x JLM → Dictator Game | -0.86 (0.39, 0.03) | -0.95 (0.40, 0.02) | No |
| Smiling Synchrony x Mean Smiling x JLM → Dictator Game | -1.67 (0.75, 0.03) | -1.78 (0.77, 0.02) | No |

*Note*. Only relevant regressors are shown. *SE*: standard error.

**Study 2: Closeness, Warmth, Competence, Friendship Potential, and Dictator Game After Excluding Familiar Individuals**

The robustness check of the key partner ratings and dictator game results in Study 2 confirmed our findings (Table S4). Most effects remained consistent after excluding familiar dyads, with both JLM and Gazing continuing to show stronger effects than Eyes Closed in increasing closeness, warmth, and friendship potential. However, the competence effect for JLM (> Eyes Closed) no longer reached the significance threshold ($p$ = 0.07 in the updated analysis vs. $p$ = 0.03 in the original). Additionally, JLM was no longer significantly more impactful than Gazing in increasing friendship potential ($p$ = 0.03 to $p$ = 0.09). Given that effect sizes in the original and updated analyses were nearly identical, these changes may have been due to reduced statistical power. All other effects remained significant and comparable to the initial analysis.

**Study 2: HR Synchrony Results After Excluding Familiar Individuals**

The key results for HR synchrony were robust (Table S5). HR synchrony for JLM pairs remained significantly higher than for both JLM pseudo pairs and Eyes Closed pairs. Changes occurred in the Gazing condition: its effect relative to pseudo pairs weakened (becoming non-significant), while its contrast with Eyes Closed pairs strengthened (becoming significant). These findings suggest that HR synchrony for JLM is driven by the nature of the interaction rather than familiarity. For Gazing, the change in significance may reflect an influence of familiarity or reduced sample size and statistical power. Future studies should explore this further. Importantly, given the small number of familiar individuals, no definitive conclusions can be drawn about familiarity's influence on synchrony—this analysis merely served as a robustness check.

**Table S4. Partner Rating Analysis After Excluding Familiar Individuals in Study 2**

| Outcome Variable | Predictor | Original Estimate (*SE*, *p*) | Updated Estimate (*SE*, *p*) | Change in Significance |
|---|---|---|---|---|
| Closeness | Gazing (> Eyes Closed) | 0.57 (0.15, < 0.001) | 0.55 (0.16, 0.001) | No |
| | JLM (> Eyes Closed) | 0.97 (0.16, < 0.001) | 0.95 (0.17, < 0.001) | No |
| | Pre-Exercise Value | 0.79 (0.06, < 0.001) | 0.79 (0.09, < 0.001) | No |
| | Gazing x Pre-Exercise Value | 0.07 (0.09, 0.47) | -0.03 (0.14, 0.84) | No |
| | JLM x Pre-Exercise Value | 0.00 (0.08, 0.98) | -0.10 (0.16, 0.52) | No |
| | JLM (> Gazing) | 0.40 (0.15, 0.008) | 0.40 (0.16, 0.02) | No |
| Warmth | Gazing (> Eyes Closed) | 0.20 (0.09, 0.04) | 0.22 (0.10, 0.03) | No |
| | JLM (> Eyes Closed) | 0.30 (0.09, 0.002) | 0.29 (0.10, 0.004) | No |
| | Pre-Exercise Value | 0.87 (0.05, < 0.001) | 0.88 (0.05, < 0.001) | No |
| | Gazing x Pre-Exercise Value | -0.05 (0.07, 0.49) | -0.05 (0.08, 0.52) | No |
| | JLM x Pre-Exercise Value | -0.22 (0.07, 0.003) | -0.25 (0.08, 0.002) | No |
| | JLM (> Gazing) | 0.10 (0.09, 0.26) | 0.08 (0.10, 0.44) | No |
| Friendship Potential | Gazing (> Eyes Closed) | 0.17 (0.06, 0.01) | 0.18 (0.07, 0.008) | No |
| | JLM (> Eyes Closed) | 0.30 (0.06, < 0.001) | 0.30 (0.07, < 0.001) | No |
| | Pre-Exercise Value | 0.97 (0.04, < 0.001) | 0.97 (0.04, < 0.001) | No |
| | Gazing x Pre-Exercise Value | -0.03 (0.06, 0.53) | -0.02 (0.06, 0.76) | No |
| | JLM x Pre-Exercise Value | -0.17 (0.06, 0.002) | -0.19 (0.06, 0.002) | No |
| | JLM (> Gazing) | 0.13 (0.06, 0.03) | 0.11 (0.06, 0.09) | Yes |
| Competence | Gazing (> Eyes Closed) | 0.12 (0.07, 0.11) | 0.12 (0.08, 0.12) | No |
| | JLM (> Eyes Closed) | 0.16 (0.07, 0.03) | 0.15 (0.08, 0.07) | Yes |
| | Pre-Exercise Value | 0.98 (0.04, < 0.001) | 0.99 (0.04, < 0.001) | No |
| | Gazing x Pre-Exercise Value | -0.13 (0.06, 0.03) | -0.15 (0.06, 0.01) | No |
| | JLM x Pre-Exercise Value | -0.18 (0.06, 0.001) | -0.22 (0.06, < 0.001) | No |
| | JLM (> Gazing) | 0.04 (0.07, 0.58) | 0.02 (0.08, 0.76) | No |
| Dictator Game | JLM (> Eyes Closed) | 0.56 (0.24, 0.02) | 0.57 (0.18, 0.02) | No |
| | JLM (> Gazing) | 0.30 (0.23, 0.19) | 0.29 (0.23, 0.20) | No |
| | Gazing (> Eyes Closed) | 0.26 (0.23, 0.27) | 0.28 (0.23, 0.24) | No |

*Note*. All reported results, except for "JLM (> Gazing)," refer to analyses where "Eyes Closed" was set as the reference condition. "JLM (> Gazing)" refers to the JLM regressor in models where "Gazing" was the reference condition. “Pre-exercise value” refers to the reported value on the respective outcome variable prior to starting the meditative exercise. *SE*: standard error.

**Table S5: HR Synchrony Results After Excluding Familiar Participants**

| Comparison | Original Manuscript | After Excluding Familiar Dyads | Change in Significance |
|---|---|---|---|
| JLM Pairs vs. Pseudo Pairs | $t(15346) = 4.05$, $p < 0.001$ | $t(15337) = 3.61$, $p < 0.001$ | No |
| JLM Pairs vs. Eyes Closed Pairs | $t(149) = 3.90$, $p < 0.001$ | $t(135) = 4.05$, $p < 0.001$ | No |
| Gazing Pairs vs. Pseudo Pairs | $t(12421) = 1.98$, $p = 0.048$ | $t(12418) = 1.50$, $p = 0.13$ | No longer significant |
| Gazing Pairs vs. Eyes Closed Pairs | $t(140) = -2.53$, $p = 0.01$ (*n.s.* after correction) | $t(132) = -2.71$, $p = 0.008$ | Now significant after correction |
| JLM Pairs vs. Gazing Pairs | $t(167) = 1.38$, $p = 0.17$ | $t(155) = 1.48$, $p = 0.14$ | No |
| Eyes Closed Pairs vs. Pseudo Pairs | $t(8131) = -0.50$, $p = 0.62$ | $t(8126) = -1.12$, $p = 0.26$ | No |

*Note*. Reported *p*-values are un-corrected. *n.s.*: not significant.

## Overall Conclusion of the Robustness Analysis

These analyses demonstrate that our results remain robust after excluding participants familiar with their meditation partners. This strengthens confidence in the validity of our conclusions, as the key effects persist among participants who were unfamiliar before the meditation.

**Online Resource 9: Group Composition for Study 1**

**Table S6. Group Composition.**

| | Gazing<br>$N = 28$ | JLM<br>$N = 27$ | *p* overall |
|---|---|---|---|
| Gender | | | 0.701 |
| female | 21 (75.0%) | 18 (66.7%) | |
| male | 7 (25.0%) | 9 (33.3%) | |
| Age | 25.2 (9.38) | 21.8 (4.05) | 0.081 |
| Race | | | 0.378 |
| American Indian or Alaska Native | 1 (3.57%) | 0 (0.00%) | |
| Asian | 13 (46.4%) | 10 (37.0%) | |
| Asian and other | 0 (0.00%) | 1 (3.70%) | |
| Black or African American | 3 (10.7%) | 7 (25.9%) | |
| Other | 1 (3.57%) | 0 (0.00%) | |
| White | 10 (35.7%) | 8 (29.6%) | |
| White and Asian | 0 (0.00%) | 1 (3.70%) | |
| Meditation experience | | | 0.866 |
| Never or almost never | 14 (50.0%) | 15 (55.6%) | |
| Often (a few times each week) | 1 (3.57%) | 1 (3.70%) | |
| Rarely (less than once a month) | 10 (35.7%) | 7 (25.9%) | |
| Sometimes (a few times each month) | 3 (10.7%) | 4 (14.8%) | |
| Relationship status | | | 1.000 |
| Casually dating | 2 (7.14%) | 3 (11.1%) | |
| Committed relationship(s), not married | 7 (25.0%) | 8 (29.6%) | |
| Committed relationship(s) (not married) | 1 (3.57%) | 0 (0.00%) | |
| Married | 1 (3.57%) | 0 (0.00%) | |
| Single and not dating | 17 (60.7%) | 16 (59.3%) | |

**Online Resource 10: Group Composition for Study 2**

**Table S7. Group Composition.**

| | Gazing<br>$N = 35$ | JLM<br>$N = 33$ | Eyes Closed<br>$N = 30$ | $p$ overall |
|---|---|---|---|---|
| Gender | | | | 0.462 |
| Female | 21 (60.0%) | 24 (72.7%) | 18 (60.0%) | |
| Male | 14 (40.0%) | 9 (27.3%) | 12 (40.0%) | |
| Age | 21.3 (4.59) | 21.5 (6.38) | 21.3 (7.10) | 0.991 |
| Race | | | | 0.373 |
| Asian | 19 (54.3%) | 19 (57.6%) | 16 (53.3%) | |
| Black or African American | 5 (14.3%) | 1 (3.03%) | 5 (16.7%) | |
| Other | 1 (2.86%) | 0 (0.00%) | 0 (0.00%) | |
| White | 10 (28.6%) | 13 (39.4%) | 8 (26.7%) | |
| White, Asian | 0 (0.00%) | 0 (0.00%) | 1 (3.33%) | |
| Meditation experience | | | | 0.247 |
| | 0 (0.00%) | 0 (0.00%) | 1 (3.33%) | |
| Never or almost never | 12 (34.3%) | 17 (51.5%) | 16 (53.3%) | |
| Often (a few times each week) | 1 (2.86%) | 1 (3.03%) | 2 (6.67%) | |
| Rarely (less than once a month) | 14 (40.0%) | 12 (36.4%) | 5 (16.7%) | |
| Sometimes (a few times each month) | 7 (20.0%) | 3 (9.09%) | 6 (20.0%) | |
| Very often (daily) | 1 (2.86%) | 0 (0.00%) | 0 (0.00%) | |
| Relationship status | | | | 0.250 |
| | 0 (0.00%) | 0 (0.00%) | 1 (3.33%) | |
| Casually dating | 4 (11.4%) | 8 (24.2%) | 2 (6.67%) | |
| Committed relationship(s) (not married) | 8 (22.9%) | 9 (27.3%) | 12 (40.0%) | |
| Married | 1 (2.86%) | 1 (3.03%) | 0 (0.00%) | |
| Single and not dating | 22 (62.9%) | 15 (45.5%) | 15 (50.0%) | |

**Online Resource 11: Comments by Participants**

Comments on the exercises are shown in Tables S8 and S9 below. These include all comments about the exercises reported by participants, excluding those purely about technology or logistics. Some participants did not provide a comment.

**Table S8. Comments from Study 1 (virtual)**

| Gazing | JLM |
|---|---|
| ● Interesting.<br>● it was a bit awkward at times but overall an interesting experiment<br>● I felt that the exercises were a bit redundant but otherwise pretty great. I didn't feel to much but it was a little strange just staring at someone else's face for so long.<br>● I felt a bit anxious because staring at a person for 2 minutes felt longer than it was. I also felt a bit \fidgety\ during the experiment at times, but I'm glad that it was not too long.<br>● This experiment was interesting. I thought it would be uncomfortable but it wasn't. The 2 minutes went by very quickly.<br>● Overall, the experiment was good. It is a little uncomfortable to stare at someone else for two minutes, but it got much easier with each round.<br>● Made me sleepy- I kept yawning during the last breakout room.<br>● I felt really awkward during most of it because I wasn't sure if I should try to be warmer or happier and I felt like I should so that my partner liked me more. It was fun to see other people though.<br>● I did not like this at all. I am pretty anxious, especially in social situations, so this was not a good experience for me.<br>● I thought it was slightly awkward but an interesting experience.<br>● I think it was good. Gave me some time to live with my thoughts and only do that with no distractions<br>● Maybe try out different exercises besides staring. Good overall, easy to follow<br>● i will admit that the survey was a little awkward and uncomfortable especially since it was just 2 minutes of staring at people and not being able to talk to them.<br>● At some point I wondered if other Participants were confederates.<br>● It was interesting. it is difficult to look at one person for that long; it feels awkward. And it's a little harder to look into someone's eyes over Zoom rather than in person because you're not exactly lined up. | ● It kind of hurt my eyes to look at the same place in a computer screen for two minutes consistently<br>● It was okay. It was a little weird but I didn't really experience much.[...].<br>● it was hard to concentrate when it felt like others were not concentrating<br>● took too long<br>● no suggestions. felt awkward.<br>● [...] I thought the study was very straightforward and enjoyable.<br>● The study did not feel awkward to me. I think my reactions would have been different in person. Looking at another person through a screen didn't elicit any significant feelings in me.<br>● I thought that the experiment made me think a lot. Moreover, I thought it was easier to look back at my life rather than imagine the other participant in the given situation: for instance, he or she being in joy or pain and longing for peace. I also thought that the third situation--this person wants to fulfill her need and meet the others' need--was kind of hard to think about since it did not relate to any emotion for me.<br>● It was a little awkward to stay silent during the meditation sessions, but I'm assuming that that is the point of the study so it's okay.<br>● I felt like the experiment was a cool experience overall, but I was mostly worried about how I was perceived by others.<br>● It was interesting. The staring was slightly weird but not uncomfortable. It was also difficult to not be distracted at least slightly during the 2 min period.<br>● I found the thinking prompts not to be very stimulating or thought provoking. I didn't feel much for my opposite partners. Most of the time I was more interested in their rooms.<br>● I tried to empathize with the other person. Sometime it was challenging because you just want to smile when you are staring at another person for that long. |

| | |
|---|---|
| ● See above. Also, in one session, it seemed the other participant was doing something in another screen. I could see the light changing in the reflection of his glasses.<br>● At first, it was slightly awkward, but then I got used to it and actually enjoyed the experiment.\n\nGood points- it's kind of a neat way to look at someone and to try to figure out what kind of person they are<br>● At various times I felt antsy while sitting in place, but that was not tightly related to the meditation.<br>● I enjoyed it but something felt lost over zoom<br>● I thought today's study was very interesting. I've never done a WBL where we had to be \in-person\ via Zoom and see other participants.<br>● felt a little self conscious about the attractiveness question. otherwise felt ok<br>● It was definitely awkward at first, so I couldn't keep myself from smiling/laughing. Some people tried to keep a poker face, but I don't think I could. If my microphone was on, I would be heard XD.<br>● I liked it, would be nice in group setting.<br>● Awkward over zoom I think, in person I'm guessing results would be a little different | ● I thought it was awkward at first, but by breakout room 4 I felt pretty relaxed.<br>● it wasn't really awkward which was surprising. The first room was interesting because I was actually thinking, but after that I got kind of bored and was just staring at the people<br>● Today's study, it felt very awkward just staring at people into the screen. I felt very anxious in the beginning, but then it became more manageable by the 4th person (I was still a little anxious though). Overall wouldn't want to meditate with a complete stranger on screen again.<br>● The breakout rooms felt a little off like they weren't able to assign it right. And it was definitely a very awkward situation.<br>● I thought it was really weird at first to just stare at a computer screen of another person's face, but after a while it got better.<br>● Fun to see people.<br>● Because this experiment was virtual, I did not feel any connection to the other person when I was looking at them. Rather, I felt that I was watching a video of someone. Also, I'm not sure if we were supposed to hide our own video but I found myself looking at myself more than the other person during the 2 mins.<br>● i'm curious to see how people rated attractiveness.[...]<br>● I felt somewhat anxious throughout the experiment, but not in an overwhelming way. The good points included seeing someone that I am casual friends with in the last round, while the bad was probably the initial discomfort in the first round.[…]<br>● It was very awkward- I felt awkward answering questions about people's appearance, the rest of it was okay. |

**Table S9. Comments from Study 2 (in-person)**

| Eyes Closed | Gazing | JLM |
|---|---|---|
| • it was good to relax<br>• I think you should get to know the person before the meditation - at least the name. I dont think it felt like I never had a partner.<br>• I was very relaxed during this experiment. It was a nice break from my day. My only suggestion is to make the experiment a little bit more interactive.<br>• It was relaxing, but other than that I didn't really see the purpose to be honest.<br>• My mind was trying to calm down but it was hard to do so.<br>• It was easy to concentrate for the first round, but with all the moving it is rather distracting.<br>• I am curious because i am confused<br>• I did not really understand why there was a question of sexual attractions.<br>• let us actually interact with them<br>• It was relaxing and helped me clear my mind. I definitely got a little more comfortable and relaxed over time as well. I thought it was interesting.<br>• the experiment was a little bit boring<br>• The experiment offered a nice escape from the stresses of college. No further suggestions<br>• I initially felt very uncomfortable when I realized that my partner(s) would be rating | • It was good, weird at first but then I got into the zone<br>• It was a little awkward, but as more rounds passed it became a bit more uncomfortable. The urge to make small talk also reduced as the rounds passed.<br>• At first I felt uncomfortable with such a prolonged time making eye contact with someone I don't know. I got used to it after a while.<br>• I was initially scared because I have social anxiety, but all my partners appeared somewhat similar in personality to me so I was a lot less anxious.<br>• In the beginning I was quite uncomfortable when I saw the prompt because it would bring back memories of when I tried to do this with friends as a challenge and if it was difficult with friends I could only imagine how difficult/uncomfortable it would be with strangers. But as I went through each round, I got more and more comfortable with the situation and this lead me to think that like other uncomfortable situations maybe putting yourself through it a couple of times will make the situation no longer uncomfortable.<br>• Was a bit awkward for the first time but afterwards I got used to it. Maybe improve it by letting us look at our partner at first before it starts.<br>• I enjoyed the experiment. At first, it was pretty awkward since there's never really an situation where you stare at | • The study was great. I feel as though the amount of time spent looking at each partner was sufficient. Additionally, the questions were interesting and made me want to continue paying attention to my partner.<br>• I felt happy, as the opportunity to stare into someone's eyes with nothing but a pure, humanity-tinged thought in your mind is rare and refreshing.<br>• Like I said earlier, it was difficult to always remain focused on the statement and maintain a straight face at all times, but I think I did decently well at following instructions.<br>• I mentioned all of my points in my previous answers<br>• At first I was pretty nervous and uncomfortable with holding eye contact with someone else for 2 minutes but as the experiment went on I realized we were both in the same boat and was able to hold eye contact longer. I usually don't enjoy doing ice breaker activities with strangers so I was fairly uncomfortable but I thought the experiment was interesting.<br>• a little weird. didn't understand why we didn't have a different partner each time<br>• Looking into strangers' eyes was interesting. I've taken the class before so I kind of understood how synchrony was supposed to work, it was interesting to see it in practice. Some partners felt more comfortable than others.<br>• It was fairly uncomfortable to start but got easier as it went along.<br>• Everything was good<br>• i think it would be more effective to take the mask off |

me on a scale. However, as the rounds went on, I felt less intimidated by their responses and instead focused on myself and my breathing.

- It felt very easy and simple. The questions and activities were rather uninteresting, however.
- At times I feel like the exercise did not affect my perception of my partner. My thoughts were concerned with myself during the exercise. I did not have a lot of sleep so I'm a bit tired but meditation was good that it was restful for my mind.
- Nothing against the experiment at all! I just don't like meditating (never have) because it tends to make me more anxious. Also, as someone in a committed relationship, the questions about attraction made me uncomfortable (even though I was able to answer 0 for all).
- this was marketed as a meditation study and there were points where we meditated but they were way too short to be enjoyable and stay relaxed because there was constant moving around.
- I really enjoyed it because I got to relax a bit. I wasn't able to completely focus on my breath, though, as I had a lot of other things on my mind. Overall, I had a good time. I don't have any suggestions.

someone for this long. But as the rounds progressed, it became much more comfortable. A suggestion I have is to make the instructions more clear as I heard some people talking during the rounds when they were supposed to be quiet.

- I liked how how it was random and I did not know anyone coming in. I feel like I might know someone now from only looking at them. It was interesting and different from what I was expecting
- it was entertaining
- I felt quite uncomfortable but gradually felt ok as the experiment progressed. I'm sure it feels more comfortable for people with their masks on (like me) as I felt more concealed.
- 2 minutes felt very long
- I thought it was fine.
- It was uncomfortable at first but got easier.
- I really enjoyed the study. I think clarity over how long it would [take] would be good.
- At first, it was extremely uncomfortable. As the rounds progressed, I got more comfortable with it and was able to laugh/smile less. It was never comfortable, but it certainly got more comfortable over time. It was easiest with the participant who wore a mask.
- Felt weird at first, but was actually fairly relaxing
- I liked the experiment. I was nervous, but I liked it.
- I learnt that people have great eyes :D
- It was definitely uncomfortable -- to test question better I think it would be helpful to have an

- The experiment is definitely something I have never experienced before. It reminded me of an article I read about staring into someone's eyes for 7 minutes or something will make them fall in love with you. This experiment was very interesting and I liked that I didn't already know anyone, so I could be completely unbiased. For some questions that I certainly related to more than others, I found myself to be more open to trying to understand my partner's eyes and reactions to the statement.
- I would do less rounds and have a greater variety of positive/negative feelings attached to the statements.
- It said meditation in the description, so I was expecting longer breathing exercises and not frequent starting and stopping/
- was weird having no interaction with other participants other than looking at each other.
- I enjoyed "meeting" new people.
- I think maybe having a timer somewhere to see how much time is left in each round
- Good: was novel, prompts were relatable and human, instructions were clear
- Bad: not sure if this was inevitable but some interactions were much more awkward than others
- As someone who is already sleep deprived and stressed 24/7, this exercise made me want to fall asleep at some points, but at other points made me nervous that my partner would get a bad impression of me because I am in a sad mood today so I tried my best to smile throughout all of the rounds

- I honestly learned a lot about myself- throughout the survey, I learned that I am quite an anxious person. Overall, I thought it was pretty fun though!
- I felt anxious as the experiment went on because I am not really used to meditating.
- It was nice, I enjoyed a few moments of quiet!
- I thought it was helpful in allowing me to spend some time thinking about life. However, I think the moving around can be kind of distracting and gives participants like a purpose for the next thing which can cause them to not pay attention.
- Its a bit tedious
- The experiment was relaxing.
- I enjoyed the meditating portion. I thought it was nice especially in the morning. I didn't understand the point of sitting next to someone
- This was a nice study. I enjoy meditation so I enjoyed it a lot. The fluorescent lights in the room make my eyes tired. It was nice to say hi to some new people I've never met before.
- The experiment was overall a good experience. It was bit awkward sitting in silence for 2 minutes, but I got acclimated to it by the second round.
- I felt that I wanted to converse with the other person and get to know them. It felt strange to give my impression of even number of males and females in the room
- Small amounts of back pain was experienced but that is from how I slept. Otherwise my experience was more akin to refocus on other topics while not blinking.
- Of course there was slight discomfort at first with the eye contact with a stranger, but other than that, the stale air made it difficult to keep my eyes open the entire time
- For the most part I felt pretty relaxed, I liked the room environment, but sometimes during the exercises, I would feel slightly uncomfortable and would just be waiting for the exercise round to be over.
- It was fun. I think we couldve better rated people if we had spoken to them for a minute before.
- I thought it was interesting to meet people through their eyes before talking much with them or learning about them as a person. I sometimes wondered what was going through their head.
- It was surprising at first because I didn't know it was going to be a partner exercise. But it became more comfortable as we progressed and I knew everyone was doing it. Maybe it would be nice to know each other's names before we commence the staring :)
- It was uncomfortable to look into random strangers eyes. I felt like no one knew how to act.
- i felt pretty neutral throughout except when i
- It was really awkward but fun in a way
- I really liked the study but felt sometimes that the sentences were hard to distinguish, often I wasnt as focused on the sentence itself as much as I was focused on seeing the other person as a human
- The overwhelming experience I felt throughout the rounds was that it was a little bit of a shame that some of my partners and myself for some rounds couldn't stop ourselves from laughing out of awkwardness. After the first few rounds, we were able to keep it together.
- It was really awkward but I enjoy this sort of thing. i think it is interesting how humans struggle so much with something like this which seems very base. It feels as though no one really wants to be seen by others literally and metaphorically, especially by strangers. Something of people's self doubts come to light if others look at them in this manner so it becomes very awkward and laughing becomes more of an avoidance mechanism. I found it very interesting to participate in
- I have never done an exercise like this before. I thought it was really interesting and liked how I was able to feel more comfortable/relaxed with the situation as we did more rounds.
- No suggestions really. But in general, I enjoyed it, I think the first couple of rounds were difficult just getting used to the awkwardness of the situation. Didn't feel much like meditating until the end rounds
- It was fun! I think the way the anxious sliders were confused me a bit because I would slide

| | | |
|---|---|---|
| them without knowing them, so I felt drawn to the most positive responses for every person. | had my best friend as a partner for one round. that may have skewed results depending on what is being measured<br>● I felt pretty neutral towards the activity. I noticed myself getting more used to the discomfort/the activity as the rounds progressed.<br>● '- it was an interesting experiment, it was nice that we got a break<br>● In the very beginning, the experimenters thought they didn't have my letter, so I was a little bit confused and wary that it might've been part of the experimental set-up. Thus, I kind of approached the interactions through the lens that my partners were confederates and that they had been tasked with a certain behavior to see how I would react and respond.<br>● The experiment felt a bit awkward at times and the confederate variables were a bit obvious during some rounds, but overall I enjoyed the experience.<br>● The experiment was cool! I enjoyed it. A suggestion would be circling back to your original partner at the end of the exercise to see if answers changed from before going through the rounds and after.<br>● I thought the experiments were well run. It was definitely slightly awkward the first time staring at someone for two minutes, but I became more comfortable with the exercise as we went on. I was pretty tired, so I almost fell asleep once or twice. | to the left for not relaxed but slide right for anxious. It confused me a bit.<br>● it was fun for me because i like eye contact and i like the potential of people falling in love with me<br>● Some parts of the experiment were a little awkward but overall everything ran smoothly.<br>● I enjoyed it. A bit awkward at first but I thought it was interesting because when thinking about the prompts, I found myself almost making up background stories for my partners/trying to guess what they had been through.<br>● it was a very curious experiment but i wish the lights were not dimmed as it made me excessively sleepy<br>● At first, I felt uncomfortable but as I looked into the other person's eyes I became more relaxed. I felt closer to the person after each round. It was a lot of rounds though.<br>● It was a bit awkward at times having to stare into another person's eyes for 2 minutes straight. The experiment itself was pretty straightforward though.<br>● It was an interesting experience to look at strangers for such a long duration of time. I think it felt uncomfortable at first, but was better towards the end, which may have affected my feelings in the survey.<br>● It was awkward at first especially since round 2 was when we both started smiling and holding back our laughter and averted our gaze frequently. But every round afterwards was not awkward. |

**Online Resource 12: Tips for the Practical Implementation of the Exercises**

In a practical in-person setting, the exercises can be organized in a rotational format. One group of participants remains seated, while the other group moves from one partner to another at the sound of a timer. Alternatively, if all participants are physically mobile, they can walk around a room, and you can choose to play music. When the music stops or a timer sounds, participants pair up with the nearest individual to engage in the exercise. An experimenter reads the sentence prompts to be contemplated. In a virtual environment, participants can be assigned to breakout rooms in pairs, with a timer function to manage the duration of each exercise. Be mindful of accessibility needs, particularly for elderly participants or individuals with disabilities.

If there are concerns regarding increased romantic or sexual attraction (e.g., in the workplace), you could consider conducting exercises among peers at the same hierarchical level. You could also limit them to same-gender groups in professional settings —although romantic feelings could still arise for individuals attracted to the same gender. In certain contexts, you could consider keeping the duration relatively short to mitigate the development of more intense interpersonal effects like romantic attraction. However, it is worth noting that in the current study, effects on closeness were comparable across same- and mixed-gender dyads, and participants in mixed-gender dyads did not report significantly more discomfort. Moreover, a central aspect of the JLM exercise is to transcend roles, hierarchies, and external characteristics—suggesting that applying the JLM method across such boundaries may be especially impactful.

To support participant comfort further, reassure them that sustained eye contact is not mandatory. If it feels overwhelming, they are encouraged to stay connected by gently resting

their gaze on a more comfortable area—such as their partner's hands, hair, or another neutral point.

Finally, for contexts where the awkwardness is so pronounced that it causes issues or participants are unwilling to take part, alternative exercises involving different types of interaction could be more suitable. For instance, the "*Fast Friends"* procedure, which involves reciprocal self-disclosure between strangers (9,10), or contemplative dyad meditation (4), which involves verbalizing one's present-moment experience to a listener, might be more appropriate. Participants may also be asked to verbally share their emotions about past events from their lives, which has also been shown to enhance closeness (11). Such exercises not only foster connection but also involve some activity or interaction, which is more natural. Regardless of the specific exercise employed, our findings underscore the potential of using simple dyadic exercises to foster interpersonal connection.

**Online Resource 13: Unstandardized Betas**

In Study 1, standard deviations of pre-exercise ratings were smaller than in Study 2. Therefore, the standardized betas–which are expressed in units of standard deviations of pre-exercise ratings–are somewhat larger in Study 1 than Study 2. However, the opposite was true for unstandardized betas which were expressed in original scale units (Figure S1).

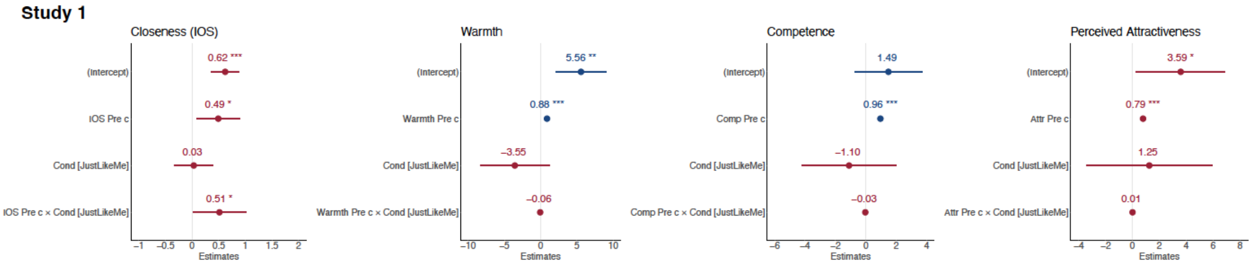

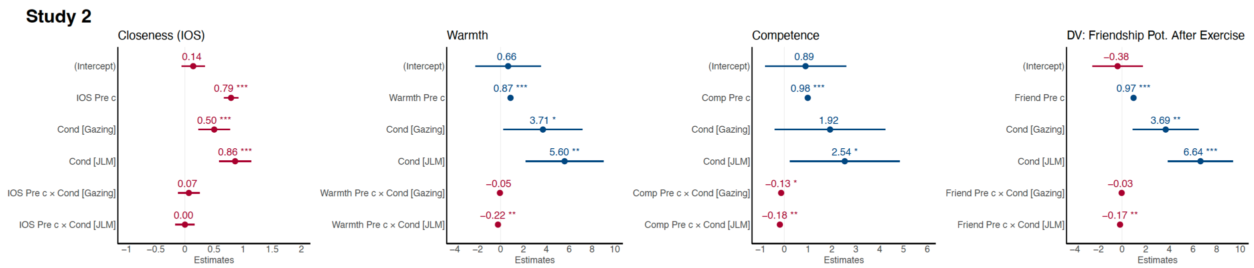


**Figure S1. Non-standardized beta values for main models.** Results from the mixed-effects model. Values in the figure are the non-standardized beta values in original units. Attraction not shown here for Study 2, as these values were only obtained from participants retrospectively. *: $p < 0.05$, **: $p < 0.01$, ***: $p < 0.001$. When comparing across studies note that the reference condition for Study 1 was Gazing, whereas the reference condition for Study 2 was Eyes Closed.

## Supplemental References


1. Stawski RS. Multilevel analysis: An introduction to basic and advanced multilevel modeling. 2013;

2. Champely S, Ekstrom C, Dalgaard P, Gill J, Weibelzahl S, Anandkumar A, et al. Package ‘pwr’. R package version. 2018;1(2).

3. Cohen J. Statistical power analysis for the behavioral sciences. 2nd ed. New Jersey, NJ: Lawrence Erlbaum Associates; 1988.

4. Kok BE, Singer T. Effects of contemplative dyads on engagement and perceived social connectedness over 9 months of mental training: A randomized clinical trial. JAMA Psychiatry. 2017 Feb 1;74(2):126–34.

5. Kuznetsova A, Brockhoff PB, Christensen RHB. lmertest package: tests in linear mixed effects models. J Stat Softw. 2017;82(13):1–26.

6. Lopes G, Bonacchi N, Frazão J, Neto JP, Atallah BV, Soares S, et al. Bonsai: an event-based framework for processing and controlling data streams. Front Neuroinformatics. 2015 Apr 8;9:7.

7. Yan W-J, Wu Q, Liang J, Chen Y-H, Fu X. How fast are the leaked facial expressions: The Duration of Micro-Expressions. J Nonverbal Behav. 2013 Dec; 37(4):217–30.

8. Shen X, Wu Q, Fu X. Effects of the duration of expressions on the recognition of microexpressions. J Zhejiang Univ Sci B. 2012 Mar;13(3):221–30.

9. Aron A, Melinat E, Aron EN, Vallone RD, Bator RJ. The experimental generation of interpersonal closeness: a procedure and some preliminary findings. Pers Soc Psychol Bull. 1997 Apr 1;23(4):363–77.

10. Cai, J., Ludwig, V.U., Platt, M.L. Creating authentic connections in virtual teams [Internet]. Knowledge at Wharton. 2023 [cited 2023 Aug 14]. Available from: https://knowledge.wharton.upenn.edu/article/creating-authentic-connections-in-virtual-teams/

11. Ludwig VU, Berry B, Cai JY, Chen NM, Crone DL, Platt ML. The impact of disclosing emotions on ratings of interpersonal closeness, warmth, competence, and leadership ability. Front Psychol. 2022 Dec 13;13:989826.
